# Potential routes of plastics biotransformation involving novel plastizymes revealed by global multi-omic analysis of plastic associated microbes

**DOI:** 10.1038/s41598-024-59279-x

**Published:** 2024-04-16

**Authors:** Rodney S. Ridley, Roth E. Conrad, Blake G. Lindner, Seongwook Woo, Konstantinos T. Konstantinidis

**Affiliations:** 1 School of Chemical and Biomolecular Engineering, Georgia Institute of Technology, Atlanta, GA 30332 USA; 2 School of Biological Sciences, Georgia Institute of Technology, Atlanta, GA 30332 USA; 3 School of Civil and Environmental Engineering, Georgia Institute of Technology, Atlanta, GA 30332 USA

**Keywords:** Data mining, Literature mining, Protein analysis, Genome informatics, Metagenomics, Biotechnology, Databases

## Abstract

Despite increasing efforts across various disciplines, the fate, transport, and impact of synthetic plastics on the environment and public health remain poorly understood. To better elucidate the microbial ecology of plastic waste and its potential for biotransformation, we conducted a large-scale analysis of all publicly available meta-omic studies investigating plastics (n = 27) in the environment. Notably, we observed low prevalence of known plastic degraders throughout most environments, except for substantial enrichment in riverine systems. This indicates rivers may be a highly promising environment for discovery of novel plastic bioremediation products. Ocean samples associated with degrading plastics showed clear differentiation from non-degrading polymers, showing enrichment of novel putative biodegrading taxa in the degraded samples. Regarding plastisphere pathogenicity, we observed significant enrichment of antimicrobial resistance genes on plastics but not of virulence factors. Additionally, we report a co-occurrence network analysis of 10 + million proteins associated with the plastisphere. This analysis revealed a localized sub-region enriched with known and putative plastizymes—these may be useful for deeper investigation of nature’s ability to biodegrade man-made plastics. Finally, the combined data from our meta-analysis was used to construct a publicly available database, the Plastics Meta-omic Database (PMDB)—accessible at plasticmdb.org. These data should aid in the integrated exploration of the microbial plastisphere and facilitate research efforts investigating the fate and bioremediation potential of environmental plastic waste.

## Introduction

The anthropogenic issue of plastic waste is widespread throughout the environment. 460 million tons (MT) of plastic were produced in 2019 alone^1^. In this same year, 353 MT of plastic were discarded, a quantity expected to triple by the year 2060^2^. Of all plastics produced each year, approximately 9% are captured and recycled for reuse; the remaining plastics are either disposed of in landfills or end up in unknown locations across the environment^3^. Recent studies have observed microplastics in highly remote regions across the earth, from the polar ice caps^4,5^ to remote mountainous regions^6^. Plastic and its additives have been reported to have potentially deleterious effects on biology in numerous studies, including perturbation of the photosynthetic activity of *Prochlorococcus* in the oceans^7^ as well as various diseases in birds^8^ and fish^9^. Predicted rates of plastics degradation vary widely, with studies such as Chamas et al. predicting rates up to 1000 years, based on 25 degradation studies available at the time of publication^10^. Additional studies have looked to further describe the characteristics of marine plastic degradation based on physical and chemical polymer properties^11^. Efforts to increase capture and recycling of plastics are underway via mechanical, chemical, and biological means^12,13^. As the production of plastics is rapidly increasing however, further understanding of the ecological response and breakdown of plastics in situ is pertinent to our response for this growing issue.

Plastics are known to degrade in the environment by various factors such as mechanochemical, photo-oxidation, thermo-oxidation, hydrolysis, and biological degradation^10^. Microbial communities are of particular significance, as these organisms are the primary means of reintroducing these polymers into the global carbon cycle via biotransformation into CO_2_ and biomass^14^. Microbes already orchestrate the biotransformation of natural polymers including complex polysaccharides such as lignocellulose^15^. Aside from the degradable class of ‘bioplastics’, synthetic polymers are generally considered highly recalcitrant materials. Literature on the microbial degradation of plastics has nonetheless increased rapidly in recent years, due increased research efforts searching for evidence of microbial evolution that utilizes the newly available manmade compounds littering their habitats. Microbial isolates have recently been reported to break down plastics such as PE and PET within several months^16,17^. Enzymes isolated from these species have been shown to degrade these plastics within hours, as in the case of the engineered enzyme FastPETase^18^, deriving from PETase found in *Ideonella sankesis.* Database compilations of these isolate studies have been assembled in efforts such as PlasticDB^19^ and PAZy^20^. However, it is unclear whether these laboratory cultures and enzymes truly represent the natural microbial communities acting on plastics in situ.

Meta-omics has been a well-utilized tool towards understanding the microbial ecology of plastics. Much of this work has been performed at the 16S rRNA gene (or simply 16S) ‘meta-barcoding’ level, allowing for insights into the community structure associated with plastics. These studies have confirmed that plastics do cause shifts in the local microbial community, which are distinct from those associated with biofilm formation on non-plastics^21^. The use of whole-genome meta-omics data, such as metagenomics, metatranscriptomics, and metaproteomics, has only recently begun to be utilized towards understanding these shifts. These data are particularly useful for studying the process of plastics breakdown, as the corresponding pathways are not well understood and, in general, are not well represented by 16S data^22^. Whole genome meta-omic data can provide a more accurate representation of the functional profile of a community^22^, as it contains the entire enzymatic profile of a given community. Microbial species are known to vary widely in their accessory gene content based on their local envrionment^23^. Therefore, observing enzymatic potential and regulation of microorganism specifically on plastics is pertinent to understanding the issue of current interest.

A growing body of recent whole-genome meta-omic studies have been performed in several environments across a breadth of plastic types, giving snapshots into the world of microbial responses to anthropogenic plastic waste. These data can enable a better understanding of microbial interactions with these pollutants, yet there is a significant gap in terms of meta-analyses exploring the broader relationship between plastics and the functional response of their associated microbial communities (i.e., the plastisphere). Recent efforts have been made towards this aim with a portion of the available metagenomic data^24^, however these lack most of the available oceanic and river plastic-associated metagenomes; these locations are a primary destination for mismanaged plastic waste^25^. Additionally, these studies do not consider the available plastic degrading isolate genomes or other forms of ‘omic data such as transcripts or proteins, which are crucial to understanding how laboratory-based bioremediation efforts compare to the microbial communities in situ. Of additional interest is whether plastics act as advantageous hosts for pathogenic microbes or virulence genes on their surface^26^. Thus, in an effort to create a more comprehensive picture of the microbial plastisphere, we have compiled all publicly available meta-omic, isolate genome, and enzyme data relating to plastics in the environment. This dataset consists of over 6 terabases of sequence data and is the largest meta-omic analysis of plastics to date to the best of our knowledge. We additionally employ statistical learning methods utilizing biointeractions towards the discovery of novel plastic degrading enzymes. Through this analysis, we aimed to gain a better understanding of how microbes are functionally responding to and degrading plastics in the environment. This manuscript provides novel means for direct enzymatic discovery from environmental data, as well as candidates for directed lab efforts in isolation of novel microbes to degrade plastics.

## Results

### Study dataset

**Figure 1:**
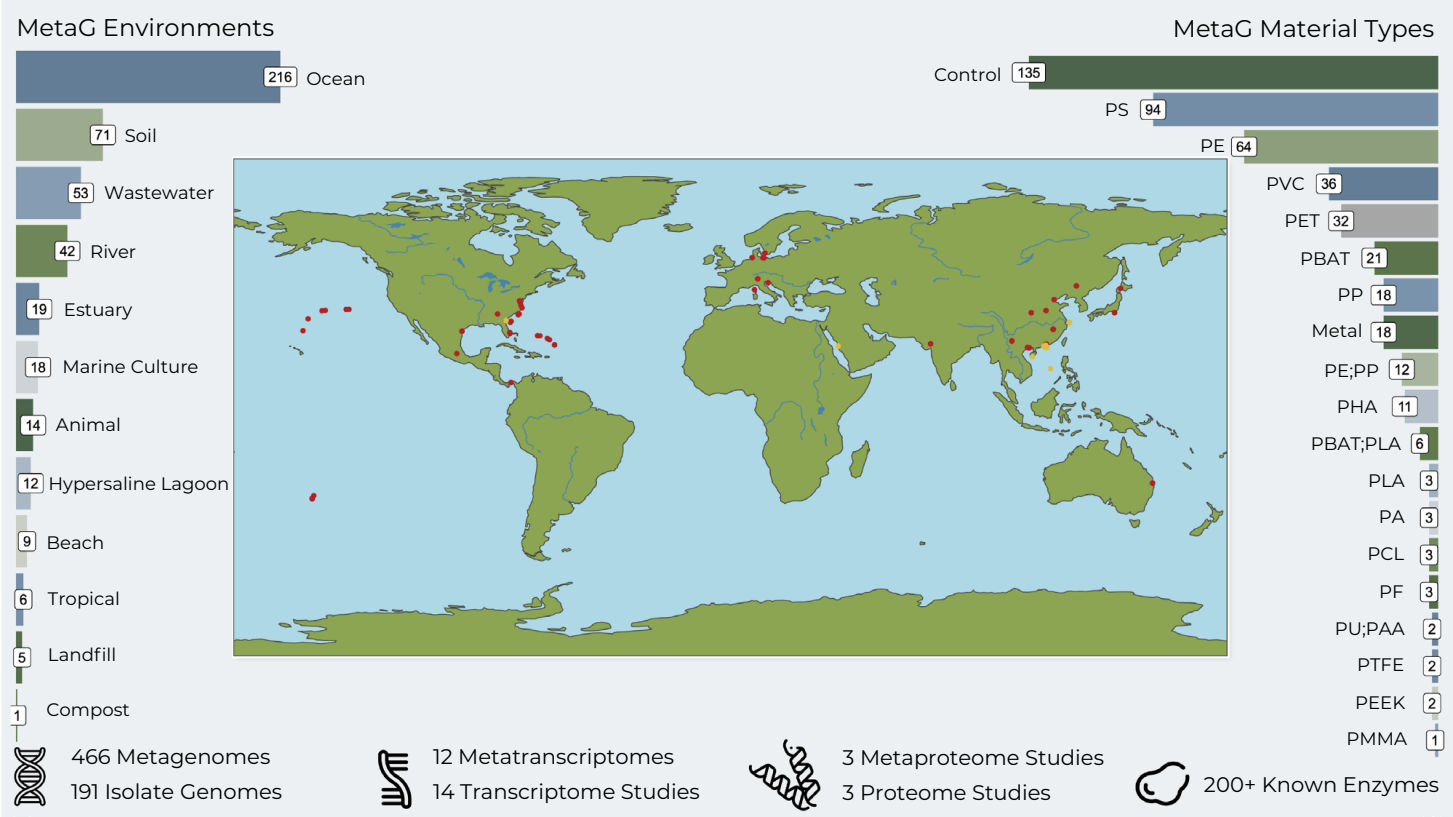
Overview of the data used in this study. Barplots and map show data for metagenomic sample locations and associated material types. Red dots on map indicate metagenomic sample was assembled via the custom metagenomic pipeline, yellow dots indicate samples from Zheng et al. biofilm study which were not assembled, but metagenome assembled genomes were instead taken from the OceanDNA catalog for the sample. Bottom area shows information about the plastisphere data which was collected from the literature. Material type abbreviations: *PS* polystyrene, *PE* polyethylene, *PVC* polyvinyl chloride, *PET* polyethylene terephthalate, *PBAT* polybutylene adipate terephthalate, *PP* polypropylene, *PHA* polyhydroxyalkanoate, *PLA* polylactic acid, *PA* polyamide, *PCL* polycaprolactone, *PF* ecovio® FT 2341, *PU* polyurethane, *PTFE* polytetrafluoroethylene, *PEEK* polyether ether ketone, *PMMA* polymethyl methacrylate, *PAA* PolyLack® Aqua Brillante, which is a polyether-polyurethane-acrylate (PE-PU-A) copolymer.

We identified 27 available studies with publicly available whole genome meta-omic data passing initial quality checks (Table 1) in April 2023. These studies were spread across 4 continents and included most major environments such as oceanic, soil, riverine, wastewater, and estuarine environments (Fig. 1). All available studies contained metagenomic data, with three studies containing complementary metatranscriptomic or metaproteomic datasets. We also included two long-read PacBio HiFi metagenomes from wastewater and degraded wood environments, which were selected as these are common locations from which plastic degraders have previously been isolated^27^. In general, studies primarily utilized incubations under natural environmental conditions lasting several weeks to a few months. Several studies also utilized laboratory mesocosms to better control for environmental factors such as UV or mechanical weathering.

**Table 1:** Meta-omic studies included in this study.

Study title and reference included in this study	Study type	Internal study ID	Environment	DOI
Whole community and functional gene changes of biofilms on marine plastic debris in response to ocean acidification^29^	Metagenome	S01	Ocean	10.1007/s00248-022-01987-w
Plastic materials and water sources actively select and shape wastewater plastispheres over time^30^	Metagenome	S02	Wastewater	10.1007/s11783-022–1580-1
New insights into the functioning and structure of the PE and PP plastispheres from the Mediterranean Sea^31^	Metagenome, Metaproteome	S03	Beach	10.1016/j.envpol.2021.118678
Insights into plastic biodegradation: community composition and functional capabilities of the superworm (Zophobas morio) microbiome in styrofoam feeding trials^32^	Metagenome	S04	Mealworm	10.1099/mgen.0.000842
Diversity and Activity of Communities Inhabiting Plastic Debris in the North Pacific Gyre^33^	Metagenome	S07	Ocean	10.1128/mSystems.00024-16
Shotgun metagenomic data of microbiomes on plastic fabrics exposed to harsh tropical environments^34^	Metagenome	S08	Fiber	10.1016/j.dib.2020.106226
Microbial Consortiums of Putative Degraders of Low-Density Polyethylene-Associated Compounds in the Ocean^35^	Metagenome	S09	Ocean	10.1128/msystems.01415-21
Microplastics altered soil microbiome and nitrogen cycling: The role of phthalate plasticizer^36^	Metagenome	S10	Soil	10.1016/j.jhazmat.2021.127944
Integrated metagenomic and metatranscriptomic analysis reveals actively expressed antibiotic resistomes in the plastisphere^37^	Metagenome, Metatranscriptome	S11	River	10.1016/j.jhazmat.2022.128418
Selective enrichment of bacterial pathogens by microplastic biofilm^38^	Metagenome	S12	River	10.1016/j.watres.2019.114979
Synergistic biodegradation of aromatic-aliphatic copolyester plastic by a marine microbial consortium^39^	Metagenome, Metatranscriptome, Metaproteome	S15	Marine culture	10.1038/s41467-020-19583-2
Shotgun Metagenomics Reveals the Benthic Microbial Community Response to Plastic and Bioplastic in a Coastal Marine Environment^40^	Metagenome	S16	Ocean	10.3389/fmicb.2019.01252
Genomic and proteomic profiles of biofilms on microplastics are decoupled from artificial surface properties^41^	Metagenome, Metaproteome	S17	Ocean	10.1111/1462-2920.15531
Plastics select for distinct early colonizing microbial populations with reproducible traits across environmental gradients^42^	Metagenome	S18	Ocean	10.1111/1462-2920.16391
Plastisphere showing unique microbiome and resistome different from activated sludge^43^	Metagenome	S19	Wastewater, Mesocosm	10.1016/j.scitotenv.2022.158330
Viral diversity and potential environmental risk in microplastic at watershed scale: Evidence from metagenomic analysis of plastisphere^44^	Metagenome	S20	River	10.1016/j.envint.2022.107146
The plastisphere microbiome in alpine soils alters the microbial genetic potential for plastic degradation and biogeochemical cycling^45^	Metagenome	S21	Soil	10.1016/j.jhazmat.2022.129941
Exploring the Composition and Functions of Plastic Microbiome Using Whole-Genome Sequencing^46^	Metagenome	S22	Ocean	10.1021/acs.est.0c07952
Soil Type Driven Change in Microbial Community Affects Poly(butylene adipate-co-terephthalate) Degradation Potential^47^	Metagenome	S25	Soil	10.1021/acs.est.0c04850
Landfill microbiome harbour plastic degrading genes: A metagenomic study of solid waste dumping site of Gujarat, India^48^	Metagenome	S34	Landfill	10.1016/j.scitotenv.2021.146184
Elucidation of the biodegradation pathways of bis(2-hydroxyethyl) terephthalate and dimethyl terephthalate under anaerobic conditions revealed by enrichment culture and microbiome analysis^49^	Metagenome	S42	Wastewater	10.1016/j.cej.2022.137916
Degradation of Recalcitrant Polyurethane and Xenobiotic Additives by a Selected Landfill Microbial Community and Its Biodegradative Potential Revealed by Proximity Ligation-Based Metagenomic Analysis^50^	Metagenome	S43	Landfill	10.3389/fmicb.2019.02986
Soil plastispheres as hotspots of antibiotic resistance genes and potential pathogens^51^	Metagenome	S53	Soil	10.1038/s41396-021-01103-9
Deciphering the role of polyethylene microplastics on antibiotic resistance genes and mobile genetic elements fate in sludge thermophilic anaerobic digestion process^52^	Metagenome	S57	Wastewater	10.1016/j.cej.2022.139520
Marine biofilms constitute a bank of hidden microbial diversity and functional potential^53^	Metagenome	S62	Ocean	10.1038/s41467-019-08463-z
Novel nitrifiers and comammox in a full-scale hybrid biofilm and activated sludge reactor revealed by metagenomic approach^54^	Metagenome	S71	Wastewater	10.1007/s00253-016-7655-9
Novel bacterial taxa in a minimal lignocellulolytic consortium and their potential for lignin and plastics transformation^55^	Metagenome	S80	Culture	10.1038/s43705-022-00176-7
Metagenomes from WWTP and wood degrading environments	Metagenome	S82	Wastewater, Wood	Samples from our work

Briefly, we utilized a custom pipeline to generate metagenome-assembled genomes (MAGs) from all available metagenomic sequence data, including rich annotations of the resulting MAGs and the remaining unbinned contigs. As most of the available data was metagenomic data, we based our analysis primarily on this type of data. We additionally provided the same rich annotations for known plastic-degrading isolate genomes. After creating a non-redundant genomospecies set from these MAGs and isolate genomes, we mapped the available metagenomic reads to these species representatives to gather relative abundance and distribution information in the environment. We defined genomospecies as 95% average nucleotide identity (ANI) of the shared gene content between related genomes, following previous practice^28^. We also performed this same mapping on a non-redundant ‘plastisphere’ gene set (n = 50,733,637) to gain understanding on the functional level of the corresponding microbial communities. Using big-data dimensionality reduction techniques, we subsequently integrate the enzymes from these metagenomic data with available non-ribosomal RNA and proteomic data in order to search the available protein space for novel plastic degrading enzymes. Further details on data collection and methodology may be found in the Materials and Methods section.

### Plastic degrading microbes span the bacterial tree of life

**Figure 2:**
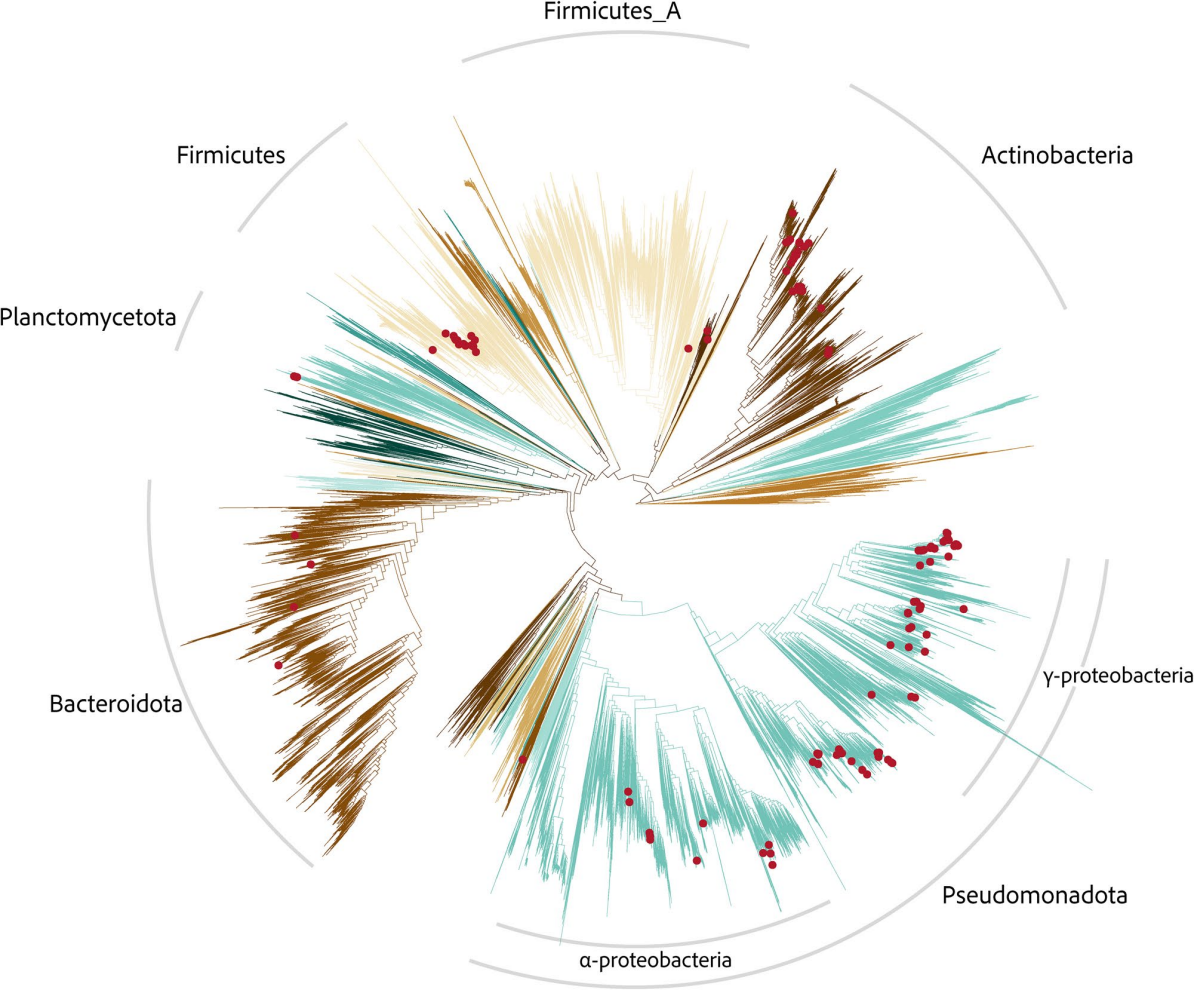
Phylogenetic tree of bacterial genomes in the current dataset. The phylogenetic tree was built using GTDB-tk in de-novo mode; only branches corresponding to genomes in the current dataset are shown (as opposed to all genomes in the GTDB database). Branches are colored according to their assigned phylum. Selected phyla and classes of interest are annotated. Firmicutes_A and Firmicutes are separate clades which were inherited as defined by GTDB r207. Red dots indicate isolate genomes which either degraded plastic or were recovered from environmental plastic samples.

In this study, we recovered 4,708 MAGs, which are widely distributed across the tree of life (Fig. 2). Of these MAGs, 3,392 were not previously classified at the species level within the Genome Taxonomy database^56^ (GTDB r207)—GTDB was used for all taxonomy reported in this study. The average CheckM (v 1.2.1)^57^ completeness and contamination of these assembled MAGs were 82.1% and 3.4%, respectively. Within this study we also included a subset of the OceanDNA MAG catalog^58^, including all the MAGs collected from the ocean biofilms by Zhang and colleages,^53^ as well as any other genomospecies in the catalog which were not otherwise represented in this dataset at the species level.

We additionally performed a literature search for microbes known to degrade or colonize plastics (isolated from environmental plastic samples, but without confirmed degradation activity). We collected a total of 166 prokaryotic genomes, 142 of which had confirmed plastic degrading activity. Of these genomes, only 68 were previously reported in PlasticDB (as of January 2023), reflecting the rapid discovery of plastic degraders in nature. More details on these genomes may be found in Supplemental File S1.

70 out of 142 isolates with reported plastic biodegradation were assigned to class *Gammaproteobacteria*. *Pseudomonas* (class *Gammaproteobacteria*) was by far the most observed genus of bacterial degrading species, containing 31 genomes with a wide variety of plastic degrading activity. *Pseudomonas* have long been utilized for remediation of various xenobiotics^59^, and are well represented in plastic degradation as well. The next most observed group with 21 degrading genomospecies was the *Burkholderiaceae* family (class *Gammaproteobacteria*). This group includes the notable *Ideonella sankesis*^17^ capable of PET degradation, as well as many isolates capable of PHA and PE degradation. *Actinomycetes* and *Bacilli* were also well represented within the degrading genomes. These groups are also well represented in the degradation of natural complex polymers, such as lignin^60^.

Of the 16 plastic types which had multiple known degraders (polypropylene only had one), there were no material types which we observed to have monophyletic degradation activity. This suggests plastic degradation is not a lineage specific function, but likely evolves due to ecological selection in the environment, and possibly horizontal gene transfer (HGT) of the selected degradation genes. Synthetic polymers have a wide variety of natural counterparts from a variety of environments^61^, thus it is not surprising that species from various taxa are adapting to utilize plastics as a carbon source. As the cleavage of plastics into short chain hydrocarbons can often occur by only a single or few genes such as PETase or cutinase^20^, it is possible that HGT of these genes is likely frequent in situ.

### Degraded ocean plastic metagenomes show enrichment of putative novel degraders

The world’s oceans are a substantial sink for mismanaged plastic waste^25^, causing major environmental perturbations such as the Great Pacific Garbage Patch. As such, the ocean has been one of the most deeply sequenced areas of the plastic environment, comprising over half of the available sequence data collected for this study. Thus, we highlight key insights specifically related to the oceanic data below.

#### Beta diversity shows differentiation of degraded vs. non-degraded plastic-associated communities

**Figure 3:**
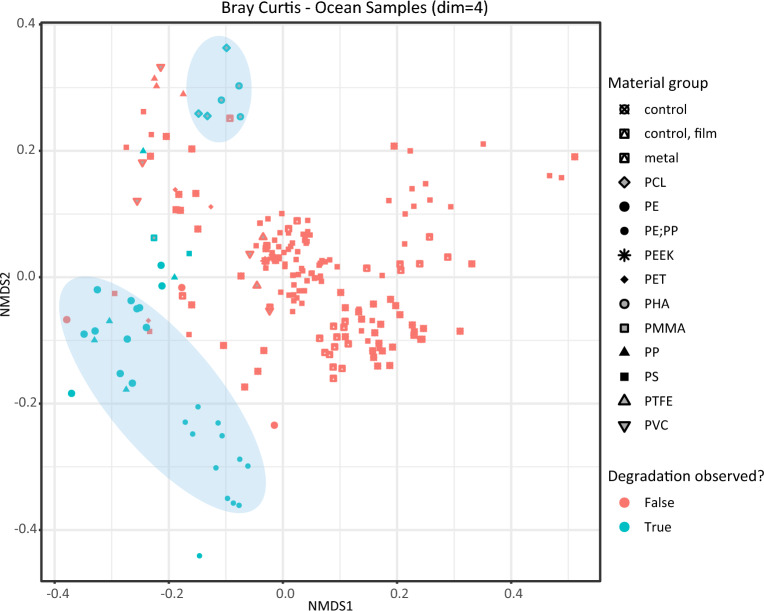
Bray–Curtis beta-diversity estimates of environmental ocean metagenomes. Blue points represent samples with degradation, while red points did not show degradation. Blue circles represent regions containing substantial clustering of degraded plastics (i.e. blue points). Figure key shows the shape which corresponds to each material group.

We calculated nucleotide level beta-diversity (i.e. how similar overall the microbial communities are among each other) estimates among the ocean samples via Simka^62^, visualizing the resulting data using Non-metric Multidimensional Scaling (NMDS). We observed clear patterns of clustering for environmentally degraded plastics vs. non-degraded samples (Fig. 3). Microbial communities on marine plastics otherwise largely clustered by material family and individual study. Metal biofilms and seawater control samples also clearly separated from plastic biofilms. Natural biofilms often were indistinguishable from polystyrene and other highly crystalline plastics, possibly connoting that microenvironments on these crystalline plastics may appear similar to other inert materials in situ. Degraded plastics formed two clear groups: one related to recalcitrant polyolefins (PE, PP) and the other to traditionally biodegradable polyesters (PCL, PHA). In the case of the PE and PP samples, quantification of the microbial effect on degradation was challenging to infer, as most of these samples were environmentally degraded without information on other important factors such as length of time or UV breakdown.

For the cluster of degraded PE and PP samples in Fig. 3, we assessed differential enrichment relative to non-degraded samples using ALDEx2^63^. We observed the differential enrichment of several genera: namely, several members of the *Rhodobacteracea* family including *Roseovarius*, *Tateyamaria*, and *JABSSA01,* along with several cyanobacterial taxa. *Roseovarius* is a group of chemoheteroorganotrophs^64^ previously observed to be enriched on plastics both in situ and under laboratory settings as a putative degrader^65^. *JABSSA01* is an uncultivated group seen previously during elevated carbon fluxes in the ocean^66^. Tateyamaria is previously known in the context of breaking down compounds used as plastic additives^67^. There are four *Rhodobacteracea* species with complete genomes currently known to degrade plastics, including a PET degrader, as well as PHA and PLA degraders. These specific genomospecies were not differentially enriched in the present samples, however.

The most enriched genus within degraded PE and PP samples relative to controls and non-degraded plastic samples was the genus *Henriciella* (effect size 0.68, wilcoxon adjusted p-value of 1.79E-04). This genus is a member of the family *Hyphomonadaceae* (class *Alphaproteobacteria*); we note this as a putative plastic degrader due to its known ability to degrade hydrocarbons^68^. We also observed that MAGs assigned to this genus across the study contained a significant number of homologs of known plastic degrading genes. The *Henriciella* group contained the highest number of such genes (n = 173) of any genus without confirmed plastic degrading isolates. Most MAGs from this genus had a common pattern of sharing four homologs to known plastic degrading enzymes. There commonly was a gene showing 53% amino acid sequence identity to both PET esterase WP_085690612.1^69^ and PLA degrading esterase AHG30919.1^70^ in *Henriciella* MAGs; these two enzymes show 21% amino acid (AA) identity to each other. There also was a repeated homolog around 58% AA identity to PEG aldehyde dehydrogenase BAF98449.1^71^. Additionally frequent were homologs to PHA dehydrogenase^72^ at 52% AA identity and PUR degrading protein ACD16728.1^73^ at 55% AA identity. Regions encoding these genes were generally spread out in different regions across the genome. More information on these genes is provided in Supplemental File S4.

For the datasets with observed plastic degradation, there was also a clear effect of sunlight, as evidenced by the location and depth of samples in the corresponding articles, as well as the abundance of *Cyanobacteria* in the datasets. It is challenging to ascertain the difference between UV (or other abiotic processes) degradation and microbial degradation, although the microbial species reported here appear to have strong potential to perform plastic biotransformation. Further culturing and experimentation would be necessary to confirm these species as plastic degraders in the ocean environment.

### Low in situ prevalence of known plastic degrading species

When looking across the environment for the genomes of species known to biodegrade plastics, we observed a somewhat surprisingly low prevalence of these organisms across the environmental metagenomes. Further, there was no correlation between the available metadata on degraded plastics and the presence or relative abundance of these degraders in the metagenomes (Adonis2, method = ’bray’). This may connote that environmental biodegradation of plastics takes place via novel microbial lineages not yet cultured in the laboratory. Surprisingly, however, we found a relatively high abundance of these known degraders in the only environmental river study available, produced by Li and colleages^44^. These samples had a strong enrichment of known degraders such as *Acinetobacter johnsonii* and *Comamonas testosteroni*, generally comprising about 1% of the total community. Despite the stark enrichment observed in our data, Li and colleagues did not include any metadata connoting that these plastics were biodegraded. Hence, to what extent plastic biodegradation occurred in their samples remains elusive. The other two major river studies available, both performed by Wu and colleages^37,38^ did show the presence of a few known degraders, such as *Azotobacter vinelandii* and *A. johnsonnii*. However, these studies did not show as strong enrichment of known degraders at the species or any higher taxonomic level as the study of Li and colleagues. It should be mentioned, however, that the metagenomes in Wu et al., studies were produced from continuous flow bioreactors of river water, rather than in situ incubations as in Li et al., which could have selected for different taxa. We observed this same trend within a set of available 16S metabarcoding studies^73–77^ from riverine environments (number of studies = 5, number of samples = 332). Plastic degraders were enriched specifically in environmental plastic incubations, but were not enriched in the set of mesocosm incubations, as shown in Supplemental Fig. S8.

We also observed the enrichment of many KEGG pathways in the river related to the degradation of various compounds associated with the degradation of plastic derivative compounds, such as styrene (map00643), caprolactam (map00930), and PAH degradation (map00624). This along with the genome level enrichment data may suggest that the river is primed for the utilization of many of the chemical compounds found in plastics. Further details on KEGG metabolism for other environments are contained in the Supplemental File S1.

### Exploring the in situ protein space for novel plastizymes

In terms of available reference genes, the known protein space for biotransformation of plastics is relatively small. For reference, at the time of this analysis there were approximately 200 genes reported to degrade plastics contained in the PlasticDB^19^ and PAZy^20^ databases. Importantly, most known plastic degrading enzymes (hereafter “plastizymes”) are capable of degrading bioplastic substrates such as PBAT, PLA, and PHB.

However, the landscape of these confirmed plastizymes is changing rapidly. Recent studies such as by Erickson and colleages^79^ have notably increased the number of enzymes and protein folds confirmed to degrade PET. Studies have also reported specifically on the distribution of PET degrading enzymes in ocean seawater^80^. Plastics such as PE, PVC, and PP still have relatively few known degrading enzymes; most of these plastics are also characterized by fairly low rates of degradation. This is likely due to the high activation energy of the polyolefinic backbones of these plastics. We additionally did find a number of recent studies claiming degradation via genes not present in either of the aforementioned databases; genes from these studies will be included in a later version of the PMDB database after further screening.

We decided to perform a de novo screening of all the data collected for this study in search of putative novel plastic degrading genes. In order to observe patterns associated with these plastizymes, we opted to assess their relative abundance and enrichment in plastic-associated samples via an amino-acid identity clustering approach, detailed in the Materials and Methods section. This analysis allowed us to gather high confidence matches for these genes and assess whether they were associated with biodegradation in the plastic environment.

#### Co-occurrence network analysis reveals coherent cluster of known and putative plastizymes

**Figure 4:**
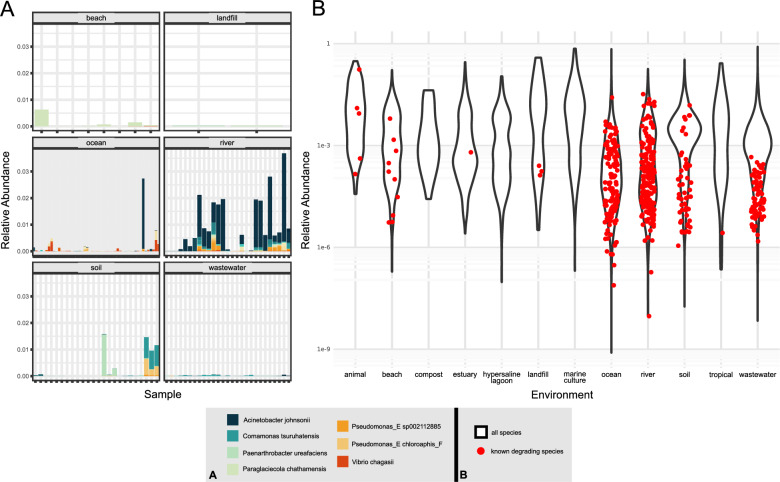
Abundance of known plastic degrading genomospecies across the plastisphere. Panel (**A**) shows the relative abundance of known degrading species in the samples within a given environment. Panel (**B**) shows the relative abundance of known degraders (red dots) compared to the abundance distribution of all species within a given environment, plotted in log scale. Abundances shown represent the 80% truncated average depth (TAD80) normalized by genomic equivalents (GEQ) in the sample, a robust metric of relative abundance (see Materials and Methods for more details). Only samples in which at least one degrading species was detected are shown. Colors corresponding to the most abundant species are shown in the figure key.

We observed a sparse distribution of known plastizymes across the plastic environment (see Supplemental Fig. S3). We observed that the most frequently detected genes were related to degradation of a nylon oligomer (caprolactam) and PHB degradation. This was not surprising, as these metabolic capacities are fairly widespread across bacteria. Notably, we observed no correlation between the presence of known plastizymes and available metadata connoting plastic degradation or the presence of plastic. This could suggest a distinction between known pathways for plastic degradation and the pathways by which degradation actually occurs in the environment, consistent with the results mentioned above at the species level (Fig. 4).

Although the known degrading genes did not follow the patterns of the available metadata, we expected that these genes would often appear in similar samples, as the corresponding populations which carry these genes were likely fulfilling a functional niche in the locations that they were observed. This was indeed the case, and we observed that these enzymes often had clusters of high Jaccard similarity in terms of their presence/absence across metagenomic samples (see Supplemental Fig. S4). The clusters observed were not limited to enzymes discovered from the same microbial species, but often clustered instead by similar chemical features of their polymeric substrates, such as polyurethane (PUR) and nylon oligomers.

As the enzymes in the environment were often observed in similar samples across the dataset, we expected other proteins with comparable functionality would also have similar observance patterns. Therefore, we utilized this sample-based correlation as a strategy for mining the data we collected in search of enzymes which could be associated with novel pathways for plastic degradation.

Uniform Manifold Approximation and Projection for Dimension Reduction (UMAP)^81^ has been a useful technique in dimensionality reduction and clustering of biological features in recent literature, having notable applications in spatial transcriptomics^82^ as well as meta-omic data studies^83^. UMAP is also well adapted for our specific dataset as many of the current correlation-based network methods are not capable of handling datasets as large as the current study, which consists of 10 + million unique protein sequences across over 400 samples. We therefore used UMAP to conduct a graph-network based analysis of the proteins across our study, selecting Jaccard similarity as the ‘distance’ metric. This framework additionally allowed us to integrate the data from other meta-omic datasets into our downstream analysis.

**Figure 5:**
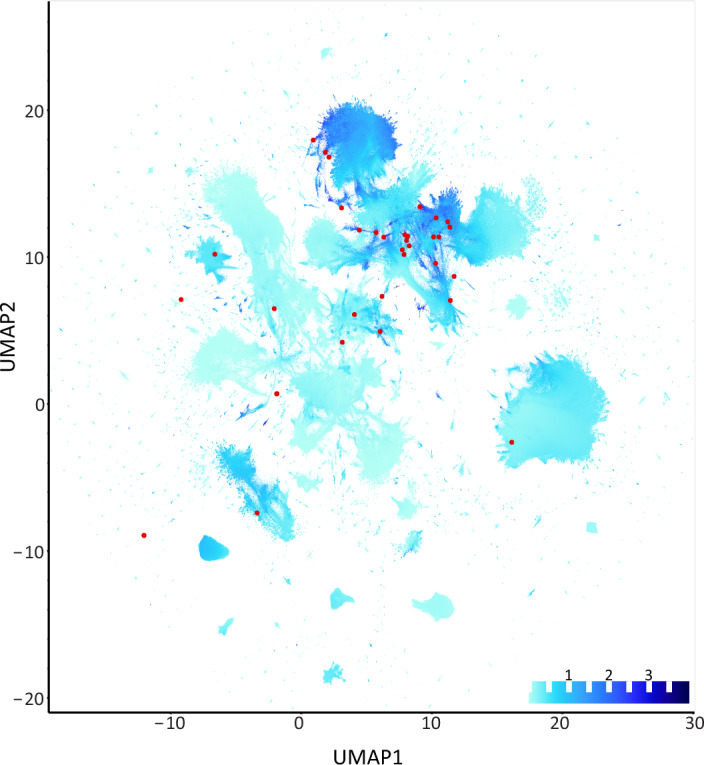
UMAP network graph for proteins observed in metagenomic ‘plastisphere’. Network embeddings are based on Jaccard distance. Nearby points therefore represent proteins of high Jaccard similarity, i.e. how often they were observed in the same samples. Points are colored based on the summation of each gene’s Bray–Curtis similarity to known plastic degrading genes in terms of presence/absence in the same samples (figure key on the right). Red dots are specific locations of proteins known to degrade plastic. Only genes present in at least nine samples are present in the plot.

The plastizymes present in the environmental metagenomes co-located into a relatively small region of the UMAP graph, connoting their strong similarity to each other in observances across samples relative to the rest of the genes analyzed. When examining the Jaccard or Bray–Curtis similarities of other genes in the dataset to the known plastizymes (Fig. 5), we observed that these genes were also commonly found in this same local space, confirming the successful embedding of these genes into this dimensionally reduced space, and indicating their putative association with plastic biodegradation. We additionally saw that the genes from the available proteomic studies consisting of proteins which were present on plastic samples were embedded in this same region. When analyzing the differentially expressed metatranscriptomes between PVC and PLA plastics, a notable portion of the most differentially expressed genes were also located in this same space (See Supplemental Fig. S5). When we examined the annotations of the genes in this region, we found they were enriched in oxidation and hydrolysis functionalities. Some of the genes of primary interest we found were WP_129973456.1—a peroxidase found in *Pseudomonas* *sp. B10* (GCF_004153525)—an organism previously isolated for PET degradation^84^, though its degradation pathway has not previously been elucidated. This gene encodes for a dye decolorizing type peroxidase (DyP)—this family of genes has been previously linked to promiscuous lignin degradation^85^. This protein family, however, has to our knowledge never been tested for plastic degradation. Notably, 13 of 14 sample observances of this gene were on plastics samples, potentially connoting the environmental selection for this gene in putative degradation environments. Another gene of interest from this space was S80_1a7091_mxb_fly_p.002 ~ BEPAJJ_15120, carried by a MAG classified as *Ochrobactrum_B sp014138095*. This MAG was abundant in the minimal lignocellulolytic consortium constructed by Rodríguez and colleagues for lignin and plastics transformation^55^. *Ochrobactrum* has previously been reported to degrade UV-treated low-density polyethylene; however, this study identified the corresponding organism(s) only at the 16S level^86^. This gene encodes for a superoxide dismutase, an enzyme previously observed to be abundant in proteomes of other plastic degraders^87^, however this gene has also not yet been specifically confirmed to degrade plastics. Finally, we found of interest the gene WP_021472099.1, a multicopper oxidase from *Paenarthrobacter ureafaciens* (GCF_002049485), a species previously reported to degrade nylon oligomers^88^. This gene was observed in nine samples across environments, primarily in river and soil samples. These genes may be tested in the future to confirm their degradation activity on plastics. We expect that this UMAP space is highly enriched with novel genes which may perform plastic degradation activity, thus we have included this UMAP information in the database which we make publicly available through this study.

### Association of plastics and microbial pathogenicity

Several recent studies have reported potential disease relatedness between plastics and pathogenic microbial species. The concern of plastic being associated with disease causing microbes has been previously connected to the ability of plastics to adsorb small molecules such as pharmaceuticals, including antimicrobials to its surface^89^. As microplastics are released into the environment by a broad range of sources which are not yet well managed^90^, there is also a concern that microbial pathogens could also adhere to these materials, and thus escape into the environment^26^. Several of these recent studies focus on antibiotic resistance genes (ARGs) as a means of studying this area of concern. An additional feature of interest would be virulence factors (VF), which would connote the potential of a microbe to infect and spread within host cells. We therefore examined the enrichments of such ARG and VF genes, identified based on AMRFinderPlus^91^ and the Virulence Factor Database^92^ (VFDB), respectively, for possible enrichment in plastic vs. control metagenomes. For groupwide comparisons, we used permutational multivariate analysis of variance (PERMANOVA) via adonis2, and differential enrichment of specific genes was assessed using ALDEx2. We did find small but significant differential enrichment of ARGs across the environment in plastic samples vs. controls (adonis2, R^2^ = 0.004, p = 0.004). Most notably, chloramphenicol resistance was differentially enriched in plastic samples across the environment, beta-lactamase resistance genes were enriched in soils, as well as other enriched ARGs in wastewater and rivers (Table 2). However, the presence of plastics connotes the presence of human pollution, thus it is difficult to confirm plastics as a direct causative factor in this increased resistance rather than common antibiotic usage by humans. In terms of virulence factors, we observed no increase in the presence of these genes between plastic samples and controls in any of the major environments. We note that these data do not specifically deny the possibility of a public health risk, as metagenomic abundance data does not truly test the infectivity of a microbial population.

**Table 2:** Differential enrichment results from ALDEx2 for antibiotic resistance genes.

Gene	Difference between (pseudo-lfc)	wilcox adj. p value
All		
Chloramphenicol efflux MFS transporter—cml	1.28	0.07
Soil		
β-lactamase oxacillinase—blaOXA	2.86	0.03
chloramphenicol efflux MFS transporter—cml	3.70	0.06
Wastewater		
Organomercurial lyase—MerB	1.26	0.08
River		
Tetracycline efflux MFS transporter—Tet (G)	4.12	0.08

Values reported are derived from either all samples or the subset of samples from the specific environment noted. Only genes with significant positive enrichment (p < 0.1, effect > 0) are shown.

### Database

In order to make the data of this study freely accessible, we have developed an online database, the Plastics Meta-omic Database (PMDB). This database contains the sequences of all proteins (91 + million) and genomes (10 + thousand) used in the study, including rich annotations as well as environmental distributions and sample metadata. Additionally included is access to the UMAP co-occurrence network graph, for interactive analysis of proteins present in meta-omic data with high probability of degrading plastics. This database presents novel access for researchers to plastic associated genomic content, as it provides information on the environmental relevance of proteins and microbial species of interest, along with rich annotations. The sequence data available also provides access for researchers for discovery of novel genes relevant to plastic degrading activity as discussed above, or analysis of pathogenic genes which may be harbored by plastics. All of these data are also fully text-searchable through the MongoDB framework. Users may additionally BLAST search their sequence of interest against the proteins in the database using PMDB BLAST. The data within PMDB is downloadable in JSON or tabular format for entire sections or search results of a database query. Further details on how to utilize this database may be found at the website (https://www.plasticmdb.org/), as well as in Supplemental File S1.

## Discussion

Plastics are xenobiotic materials which have been dispersed across the environment by mismanaged waste streams and uncaptured micro and nanoplastics. In this study, we have conducted the largest analysis of meta-omic data associated with plastics to date, documenting the in situ microbial shifts in response to these environmental pollutants. Microbes capable of utilizing these compounds are generally phylogenetically diverse, with *Pseudomonas* and *Burkholderiaceae* groups including many plastic degrading species. These known degraders were found to be very sparse in the environmental datasets analyzed here, however. The river was the main location we found known degrading species and enzymes to be relatively abundant across a wide variety of plastic types. Why would riverine systems be enriched with these known degrading species? Rivers are naturally eutrophic environments, containing many lignocellulosic compounds from plant sources. These compounds are chemically similar to plastics, thus adaptation to these new synthetic carbon sources with natural homologs should be evolutionarily feasible. Additionally, rivers are a major acceptor of land runoff, as well as anthropogenic pollution, including microplastics, when located near densely populated areas. Thus, one of the major places which plastics have likely been long available for microbes to adapt to is in riverine systems. There is also generally bioavailable oxygen in this habitat, allowing for ready incorporation of oxygen onto plastics by radical or enzymatic oxygenation. Somewhat surprisingly however, there were few isolation studies which looked to specifically isolate plastic degraders from the river. Thus, we highlight this environment as potentially crucial to in situ plastics biotransformation. We recommend riverine systems as locations of further multi-omic investigation and isolation studies for novel plastic degraders.

The ocean environment has been a deeply sequenced habitat of the plastisphere. We observed clear distinctions between plastics which had degradation and other samples that did not report plastic degradation but only plastic presence via NMDS analysis, and further distinction between the former plastic samples and free-living seawater samples. Natural biofilms did not strongly separate from non-degraded hard plastics, possibly indicating that these substrates may elicit similar responses by the microbial communities. We observed the genus *Henriciella* to be a group of high interest for future isolation and plastic degradation studies, based on its differential enrichment in degraded samples along with many homologs to known plastizymes present in the *Henriciella* MAGs recovered here. *Henriciella* and the other enriched marine taxa identified by our study have been previously associated with the flux of carbon in the environment and may be essential to understanding the biotransformation of synthetic polymers that are currently ubiquitous in the environment. Therefore, isolation and characterization of these microbes may open new insights into the ability of microbes to incorporate synthetic xenobiotics back into the global carbon cycle. Omics-based studies that target microbial activity may also be useful to elucidate these mechanisms. Metatranscriptomic and proteomic comparisons of these microbial communities between microplastics, cellulosic compounds such as laminarin, and controls may explicate how these microbes specifically respond to plastics.

Microbial data on plastics in other environments consistently showed an enrichment of specific KEGG pathways corresponding to the utilization of plastic compounds. The pathways included radical-based oxidation, together with pathways for beta-oxidation and subsequent transformation into amino acids, polysaccharides, and other forms of carbon. This schema is common across the plastic biodegradation literature and appears generally applicable across the metagenomic results obtained here as well. We would encourage follow up studies specifically investigating the generation of reactive oxygen species (ROS) by microbes to evaluate the relative contribution of this mechanism in situ*.* Studies have previously used UV pre-treated plastics to introduce radicals and subsequently oxygen onto the surface of plastics; this process has been seen to increase degradation rates, but not to levels desirable for ‘biodegradable’ plastics^93^.The ability of microbes to control the use of ROS as a method of biotransformation of synthetic polymers is an area of interest for further study.

As noted previously, there is also very few anaerobic samples within this dataset, limiting the application of these data to aerobic environments. The response of microbes to lignocellulosic compounds is largely different based on the presence of oxygen, with single enzymes being deployed to degrade these compounds when oxygen is available. However, in anaerobic environments, large multi-enzyme complexes such as the cellulosome have been observed^94^. It is possible that these differences in deconstruction pathways may be present for plastics as well.

Enzymes capable of degrading plastics are a promising biotechnological solution for bioremediation and valorization of these materials. Known plastizymes appeared sparsely in the available metagenomic dataset, with no correlation to metadata relating to plastic or biodegradation in the corresponding samples. As neither degrading genes nor genomes in situ corresponded to the available metadata, we expect that the microbial populations and enzymes performing biotransformation of plastics in situ have largely not yet been reported and are distinct from previous laboratory isolates. These enzymes did appear to correlate with one another in terms of presence or absence relating generally to the enzyme’s material substrate.

In order to search the available protein space for putative novel plastizymes, we hypothesized that previously described enzymes are the best ‘hook’ for finding novel enzymes capable of the activating the carbon–carbon and carbon-heteroatom backbones of synthetic polymers. UMAP embeddings showed the known plastizymes localized together within a dimensionally reduced protein space, connoting their strong similarity in terms of co-occurrence among all the genes in the plastisphere protein space. Proteins which were enriched in other forms of meta-omics were also frequently observed in this same region. Several promising proteins for plastic degradation from this space were reported above as well. We therefore predict this coherent sub-space to be enriched with novel degrading genes capable of performing biodegradation of plastics and encourage researchers to utilize proteins from this dataset for further testing and investigation.

Within the available data, a common limitation for the studies was the lack of additional metadata, including plastic additive information, and degradation information via robust testing methods, such as x-ray photoelectron spectroscopy (XPS) and gel permeation chromatography (GPC). Additionally, data such as microbial loads for true quantification of microbial absolute abundances limit the power of the statistical methods utilized by this study. These forms of data could be used for Canonical Correlation Analysis or further statistical learning on the data. We additionally note the lack of meta-transcriptomic and meta-proteomic studies available, which would further expand our understanding of expression resolved in situ responses to plastic at the enzymatic level. We encourage further datasets that include these forms of data to allow greater granularity on biological responses to plastics.

We observed enrichment for known degraders in riverine environments, which are known to be locations containing many human pollutants and similar polymeric compounds. 16S data has been gathered for other locations which may also contain many of these compounds, such as landfills or insect guts. This data however is fairly sparse at the whole-genome level. Increasing sequencing from these locations will add further granularity into the ability of plastics to be biodegraded in these habitats. This may also allow for more in-depth comparison of the metabolic responses to plastic in locations of enriched degradation-associated microbial communities. Polymeric materials additionally vary in degradability based on crystallinity, molecular weight, and chemical composition, alongside other factors. Studying these factors plays an important role in understanding degradation mechanisms for specific polymers. We also again note the difficulty in differentiating biodegradation from UV or mechanical degradation. Efforts to perform in situ experimentation controlling for these effects may give greater detail into the mechanism by which microbes respond to plastics in our environment. Further in-depth study may be performed in terms of how the physicochemical characteristics of different polymers effect the microbial community, as well as the various within-environment factors which may shape the ability of microbes in these habitats to degrade plastic. This data is available in the current dataset and connected database, and we encourage researchers to access these for further exploration.

## Materials and methods

### Data curation

To collect all currently available (as of April 2023) meta-omic studies relating to plastics, terms involving various major plastics and meta-omics (metagenomics, metatranscriptomics, etc.) were searched through the Web of Knowledge and Google Scholar with relevant keywords. Only papers containing publicly available data were retained. Similar search terms were used for isolate studies. Previously curated databases PlasticDB^19^ and PAZy^20^ were also parsed for studies containing complete genomes, as well as known enzymes to perform biodegradation. 16S rRNA gene (16S) data was not considered for this study, as previous papers have described this type of data in detail^21^.

For each paper retained, metadata was collected for geolocation, environment, polymer type, polymer size, and biological degradation information. Biodegradation was considered present based on what the authors reported in the corresponding manuscripts. If degradation was not specifically reported in the paper, plastics were considered to be degraded if the collected samples showed strong signs of degradation via oxidation or physical breakdown, or were otherwise well characterized biodegradable plastics kept for long incubation periods. Each study and sample was given a unique identifier, which were utilized for subsequent analysis. Study and sample metadata is available in Supplemental File S2, as well as the database provided by this paper.

### Bioinformatics pipeline for data processing

Metagenomic samples were processed using a custom pipeline developed using Snakemake (v7.16.0). Briefly, samples were trimmed using fastp (v0.23.2) with default settings, and additionally normalized using BBnorm (v38.94). For studies containing paired end reads, both trimmed and normalized libraries were assembled using metaSPAdes (v3.15.5)^95^ and IDBA-UD (v1.1.3)^96^. For large and high complexity samples, MEGAHIT (v1.2.9)^97^ was used as a second assembler instead of IDBA-UD. For single-end samples, IDBA-UD and MEGAHIT were used as assemblers. For long-read samples, metaFlye (v2.9.1-b1780)^98^ was used as an assembler. Resulting assembled contigs were filtered with a minimum length of 1 Kbp, and assembly statistics were collected using MetaQuast (v5.0.2)^99^. The trimmed reads were mapped to the assemblies using bwa-mem2(v2.2.1)^100^ and contig depths were collected using CoverM (v0.6.1) with method ‘metabat’, and otherwise default settings. Each of the four resultant assemblies was binned using MaxBin2 (v2.2.7)^101^, MetaBAT2 (v2.12.1)^102^, and Rosella (v0.4.2). For long-read studies, GraphMB (v0.1.5)^103^ was used as a fourth binner. Bin qualities were assessed using CheckM (v1.2.1)^57^ and CheckM2 (v0.1.3)^104^. Bins were dereplicated at the sample level using dRep (v3.4.0)^105^ with ANI level 95.0, S_algorithm method fastANI (v1.33)^106^ minimum completeness of 50 and maximum contamination of 10. Genome statistics were collected using SeqKit^107^. Dereplicated bins were annotated using Bakta (v1.8.1, database version 5.0)^108^, eggNOG-mapper (v2.1.9)^109^, CAZy (downloaded Aug 2022)^110^ via dbCAN2 (v3.0.7)^111^, and KEGG^112^ via KofamScan (v1.3.0)^113^. MAG taxonomy was assessed using GTDB-tk (v2.2.1)^114^.

Diversity information for each sample was also estimated: alpha-diversity was assessed using Nonpareil (v3.4.1)^115^, and taxonomic information was collected using Kraken (v2.1.2)^116^ and Bracken (v2.8)^117^. Genomic equivalents in each sample were estimated using MicrobeCensus (v1.1.1)^118^ with parameters n = 100,000,000 and q = 10.

The pipelines developed are available on GitHub at https://github.com/Rridley7/Plastic_assc_info.

For samples from the biofilm study by Zhang and colleages^53^, MAGs from the OceanDNA^58^ database were collected. Additionally included in the dataset were species representative genomes from OceanDNA which did not already have a same-species-representative in the dataset, based on a 95% nucleotide sequence identity threshold as computed by FastANI. For genomic equivalents in these metagenomic samples, reads were trimmed and assessed with the same tools and parameters as mentioned above.

For dereplication of MAGs and isolate genomes of the entire study, dRep was used with the same parameters as previously described, and sample quality information from CheckM. MAGs which passed CheckM2 but did not pass CheckM were also retained after manual checks and dereplication using skani (v0.1.4)^119^.

Beta-diversity was assessed for metagenomic reads using Simka (v1.5.3)^62^ with default settings, which calculates beta-diversity using nucleotide kmer diversity. Non-metric multidimensional scaling (NMDS) of the resulting bray–curtis distances was assessed using metaMDS from the vegan (v2.6–4) package and visualized using ggplot2 (v3.4.2)^120^.

All vs. all genome comparisons across the study were completed at the ANI and AAI level using fastANI^106^ and fastAAI^121^ respectively.

### Genome and gene mapping

Genomic and gene level abundances in the environment were assessed by mapping metagenomic reads back to non-redundant genome and gene sets. The dereplicated genome set described previously was used for the genome level abundances. Additionally, the species representatives from the OceanDNA set were utilized.

For gene level abundances, both genes carried by genomes and assembled but unbinned genes were considered. Briefly, 95% ANI genomospecies clusters from dRep^105^ were clustered using Roary (v3.13.0)^122^ with default settings. Additionally, known genes from PAZy and PlasticDB with nucleotide sequences available were added to this database of binned genes. This database was clustered using MMSeqs2 (14.7e284)^123^ at 99.9% nucleotide identity and coverage to remove duplicate genes.

To collect unbinned genes, contigs were taken from the filtered metaSPAdes, IDBA-UD, or metaFlye assembly for each sample. Genes from all contigs were predicted using Pyrodigal (v2.0.4)^124^, a python library binding to Prodigal^125^. Genes were subsequently dereplicated using MMSeqs2 at 99.9% nucleotide identity and coverage. This gene set was mapped to the binned genes using minimap2 (v2.21)^126^ using setting“—for-only”. Genes mapping to the binned set with nucleotide identity > 95% and coverage > 98% were removed from further analysis as redundant with the binned gene list. The remaining unmapped genes were subsequently clustered at 95% nucleotide identity and 98% alignment length using MMseqs2, to produce a non-redundant unbinned gene set. Unbinned genes were annotated using the same tools as described in the pipeline.

Reads from all samples were mapped to genomes and gene sequences using bwa-mem2 and CoverM using nucleotide identity > 95%, read alignment > 70%, and covered fraction > 10% as mapping thresholds. Minimap2 was used for long reads with the same settings. For the gene mapping, an iterative subtractive mapping approach was used. Briefly, reads were first mapped to binned genes, then unbinned reads were collected using SAMtools^127^. These unmapped reads were then mapped to the unbinned genes using the same parameters as previously described. The mapping pipeline is also available on GitHub at the same link as above.

Abundance at the genome level was assessed using the truncated average depth at 80% (TAD80) metric, normalized by the genomic equivalents (GEQ) estimate from MicrobeCensus within a given sample. The truncated average depth at 80% refers to the average of the sequencing depths over the indices of a feature of interest (gene, genome, or other feature), truncated to the middle 80% of those indices. This removal of bottom 10% of indices is useful for ensuring that genomes are above likely limits of detection within a metagenome and that highly variable (not conserved) regions of the genome within the population sampled are not causing the underestimation of relative abundance. The top 10% ensures they also not biased by regions which may be highly similar across various species, such as ribosomal RNA^128^. Genomic equivalents refer to the estimated number of microbial genomes within a particular sample. By dividing the TAD80, which provides a value of the number of genomes, by GEQ, which normalizes for average genome size differences among microbial communities sampled, we obtain a relative abundance metric for each microbial population within our sample. For more information on this metric, we refer readers to our recent article^128^. Gene abundances were assessed using a less stringent TAD of 90% normalized by GEQ. This metric gives similar results to transcripts per million (TPM), however GEQ accounts for more directly for the number of genomes in a metagenomic sample and their average genome size, when external microbial loads are not available.

### Genome relative abundance and phylogenetic analysis

For genomic abundance, ALDEx2 (v1.28.1)^129^ was used on samples from individual environmental subsets, to assess differential enrichment while accounting for data compositionality. Heatmaps of relative abundance were produced using ComplexHeatmap (v2.15.1)^130^, and barplots were produced using ggplot2.

Phylogenetic trees were produced via two methods. A tree comparing the genomes from the current study to the current prokaryotic tree of life was produced using GTDB-tk *in *de novo mode. Trees involving only genomes from the current dataset were produced using Phylophlan (v3.0.67)^131^. Trees were annotated and visualized using ggtree (v3.4.4)^132^ and iTOL^133^.

### Gene analysis

For analysis at the gene level, genes were clustered into high identity gene families subsequent to read mapping. The gene set containing all genes from genomes in the plastic environment, known genes, and unbinned genes was collected, consisting of 92,930,684 protein sequences. These genes were clustered using MMseqs2 at the 90%, 70% and 50% amino-acid identity levels. The 90% and 70% identity clustered required 80% coverage, while 50% was reduced to 70% coverage for lower stringency. Parameters –cov-mode 1 –cluster-mode 2 –cluster-reassign were used for all clustering. The procedure was completed in a cascading fashion, with sequences unique at higher levels being given as input to subsequent rounds of clustering. Gene cluster statistics were then collected, noting which clusters contained known degrading genes or genes from higher meta-omic (transcriptomic, proteomic) datasets. Gene statistics subsequent processing of the dataset were completed primarily using Dask^134^ for multi-threaded and larger-than-memory processing. Abundances for each gene cluster were considered by using the sum of abundances of the gene assigned to the cluster. Individual gene abundances were calculated by read mapping, using 10% trimmed mean average depth (TAD90), which accounts for gene length and edge effects when mapping to short genes, as described above.

### KEGG pathway analysis

For KEGG analysis, the top match from KofamScan annotations were used for the non-clustered dataset, with a minimum e-value threshold of 1e-5. For genes without a KofamScan annotation, KEGG annotations were collected using KEGG modules provided though UniProt annotations via Bakta. Genes without annotation after the latter step were not included in subsequent analysis. Genes were summed in KEGG modules using TAD90 values for abundances. These abundances were subsequently passed to ALDEx2 within subsets based on environment, using ‘lvha’ as the denominator. Results from ALDEx2 were sorted by effect size, and passed to clusterProfiler (v4.4.4)^135^ using method gseKEGG with parameters pvalueCutoff = 0.05, nPermSimple = 10,000, and eps = 0.

### UMAP co-occurrence network

Uniform Manifold Approximation and Projection^81^ (UMAP, v0.5.3) embeddings were produced via use of the 90% level clustered gene set, using all gene clusters observed in a minimum of 9 samples, to avoid spurious correlations. The Jaccard distance metric was used for all runs; other metrics were selected via manual tuning. The main graphs presented in this manuscript used metrics *n_neighbors* = *20* and *min_dist* = *0.3*. UMAP data was visualized using hvplot and datashader^136^, using bokeh as a backend framework^137^. Bray–Curtis similarities of metagenomic abundances to known plastic degrading enzymes were calculated using Dask (v2023.7.0) and scipy^138^. Values reported within figures are summed cumulatively across all known plastizymes for each gene.

### Virulence and AMR genes

For antibiotic resistance and virulence related genes, gene annotations for the non-redundant gene dataset were collected using AMRFinderPlus (v2022-12-19.1)^91^ and VFDB (v2023-02-10)^92^. Genes from across the dataset were annotated by Bakta as previously described—the Bakta program reports annotations from each of the above programs within its output. Gene abundances from all genes matching a specific annotation were summed as previously described. Group-wise comparisons were assessed using adonis2 in the vegan package, and differential enrichment of specific genes was tested using ALDEx2. Comparisons which were for plastics across all environments used denom = ’zero’, while individual environments used denom = ’iqlr’. Heatmaps were generated using ComplexHeatmap in R.

### Other meta-omic datasets

Data from transcriptomic and metaproteomic datasets were included in the non-redundant protein set. Genes from these sets were given numeric rankings for subsequent searchability based on the study type (proteome given higher ranking than transcriptome), and whether differential enrichment was observed in the dataset. Genes from these studies were annotated using the same methods as described previously. These numeric rankings are also included in the PMDB database.

For the meta-transcriptomic dataset published by Wu and colleages^37^, reads were first trimmed using fastp. Trimmed reads were then sorted using SortMeRNA (v2.1)^139^, to retain non-ribosomal reads. Non-ribosomal reads were mapped to a non-redundant metagenomic gene set using CoverM and bwa-mem2 using the same parameters as previously described. The non-redundant gene set was produced using metaSPAdes assembled contigs from the pipeline, dereplicated at 95% nucleotide identity and 98% coverage using MMSeqs2. Differential abundance of mapped transcripts was assessed using ALDEx2^129^, and plots were produced using ggplot2.

#### 16S metabarcoding analysis

For the analysis of riverine 16S metabarcoding studies in comparison to the available plastic degrading isolate genomes, studies containing 16S data were found via searching ‘16S river plastic metagenome’ through Google Scholar. Reads of these studies were obtained from the SRA database of NCBI, trimmed using fastp (v0.23.2) with default settings, and subsequently mapped to the known degrader isolate genomes using Magic-BLAST^140^ (v1.7.0). 16S reads were considered to be a match if at least one paired-end read mapped to a genome with higher than 98.5% nucleotide identity^141,142^ (i.e. species-level assignment). Reads with ties between multiple genomes were randomly assigned to a single genome. Relative abundance was assessed using the number of reads mapped to a specific genome normalized by the total number of reads in a sample. The analysis was completed using custom python scripts, and plots were generated using ggplot2 (v. 3.4.2). 16S samples and associated metadata are available in Supplemental File S5.

### Database

**Figure 6:**
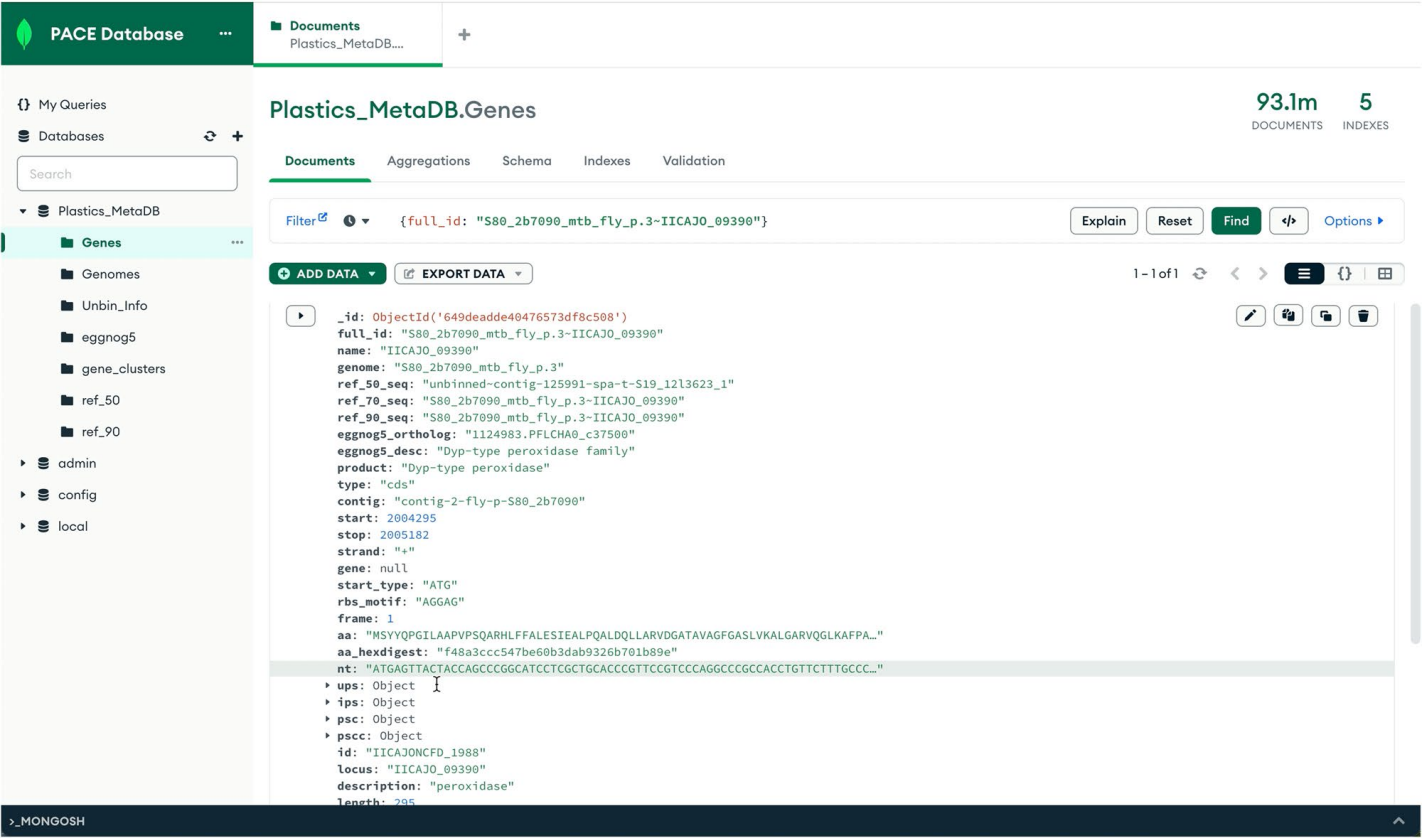
Plastics Meta-omic Database (PMDB) preview, shown in MongoDB Compass.

Study metadata, genomes, genes, and UMAP data were collected into a database using a local instance of MongoDB Community Edition (v6.0.5) via custom python scripts. There are several sections available in the database, corresponding to gene annotations, genomes, gene clusters, and study metadata previously described (Fig. 6). Sections in the database are fully searchable by annotation, name, and sequence information by text indices in MongoDB. Sample metadata and genome sequences will also be separately made available for access.

### Metagenome sampling and DNA extraction

The return-activated sludge was obtained from a wastewater treatment plant in the Atlanta Metropolitan area, Georgia, USA. Around 1 L of sludge was put into a sterile 1 L glass bottle and was transported in ice to the lab. 50 mL of sludge was aliquoted to a 50 mL conical tube and centrifuged at 4 °C, 5000*g* for 10 min. The resulting supernatant was thrown away and the pellet was used for extraction immediately. The degraded wood was sampled from a dead and decaying broad-leaved tree in the Atlanta Metropolitan area, Georgia, USA in the winter of 2022. The inside of the tree was scooped with a sterile 50 mL conical tube and the lid was closed immediately. The sample was stored at 4 °C until the extraction. The DNA was extracted with the Qiagen DNeasy PowerMax Soil kit following the manufacturer’s protocol. The quality of the extracted DNA was analyzed with Thermo Fisher Scientific NanoDrop 1000 Spectrophotometer and the quantity was accessed with Invitrogen Qubit 1X dsDNA HS assay kit and Invitrogen Qubit 3.0 Fluorometer. The checked DNA was stored at -20 °C until the sequencing.

The library preparation and sequencing were performed by the Georgia Genomics and Bioinformatics Core (GGBC) (Athens, Georgia, USA). The DNA extracts were sent to GGBC where Pacific Biosciences (PacBio) Single Molecule, Real-Time (SMRT) bell multiplex library was constructed without the shearing step and was sequenced with a single PacBio SMRT Cell on the Sequel II system.

### Supplementary Information

Supplementary Information 1.

Supplementary Information 2.

Supplementary Information 3.

Supplementary Information 4.

Supplementary Information 5.

## Data Availability

The resulting sequences and graph network data from this manuscript can be accessed through the Plastic Meta-omic Database at https://plasticmdb.org. The Snakemake pipeline code used for the metagenomic analysis may be found at https://github.com/Rridley7/Plastic_assc_info. Sequence accessions for the associated studies may be found within the manuscripts referenced in Table 1. PacBio Hi-Fi sequences from our original wastewater and degraded wood samples are available at NCBI BioProject ID PRJNA1041404.
