# Supplementary material for: Potential routes of plastics biotransformation involving novel plastizymes revealed by global multi-omic analysis of plastic associated microbes
Source: Sci Rep. 2024 Apr 16;14:8798. doi: 10.1038/s41598-024-59279-x (PMC11021508; doi:10.1038/s41598-024-59279-x)
Supplement: Supplementary file 1 — Supplementary Information 1. [file 41598_2024_59279_MOESM1_ESM.docx]

**Supplementary Information for “Potential routes of plastics biotransformation involving novel plastizymes revealed by global multi-omic analysis of plastic associated microbes”**

**Rodney S. Ridley, Jr^1,*^, Roth E. Conrad^2,3^, Blake G. Lindner^3^, Seongwook Woo^2^, Konstantinos T. Konstantinidis^2,3,*^**

^1^Georgia Institute of Technology, School of Chemical and Biomolecular Engineering, Atlanta, Georgia, 30332, United States

^2^Georgia Institute of Technology, School of Biological Sciences, Atlanta, Georgia, 30332, United States

^3^Georgia Institute of Technology, School of Civil and Environmental Engineering, Atlanta, Georgia, 30332, United States

^*^Correspondence to: [rridley3@gatech.edu](mailto:rridley3@gatech.edu) and [kostas@ce.gatech.edu](mailto:kostas@ce.gatech.edu)

**PMDB Database description**

Metadata for all metagenomic and metatranscriptomic samples contained within the dataset is included in the *Metadata* section of the database. Each record in this section contains the associated metadata manually compiled from the corresponding manuscripts, as well as associated data in the sequence archives from which sequences were originally downloaded. This metadata includes details on the environment, material type, and whether degradation was observed in a particular sample.

Each genome within the *Genomes* section of PMDB is uniquely named based on its sample of origin using the study identifiers within Table 1. Genomes from external sources such as NCBI or the OceanDNA catalog retain their names from these original sources. Each genome record includes taxonomic classification and quality information from CheckM, in addition to whether the genomospecies is a known degrader of plastic. To search for genomes which were enriched in any of the subsets mentioned in this manuscript, we will also include a section *MetaG_Genomes.* This section includes abundance information for all genomes observed in the dataset, as well as differential enrichment information from ALDEx2 for selected environmental subsets of interest.

The *Genes* section of the database contains the over 91 million unique proteins observed in the plastisphere. All proteins contained within a genome are linked by name to this genome, while unbinned proteins are linked to the sample from which they originate. Additionally included in the *Genes* section is all protein confirmed in the current dataset to degrade plastic – this subset is easily accessed by searching *is_known = True*. These known degrading proteins additionally contain citation information and details on the plastic type they degrade. Every protein within PMDB is fully text searchable by name as well as annotation information, allowing researchers to easily find all plastic-associated proteins which perform a specific function, such as hydrolases or polysaccharide lyases. Proteins are also searchable by enzyme families, such as Enzyme Commission (EC) numbers or Pfam family. Protein groups by 90%, 70%, and 50% sequence identity are also identifiable, including whether there is a protein known to degrade plastic within these limits of identity to any protein of interest.

In order to more directly search for proteins that have a high probability of degrading plastics, we additionally make available the UMAP embeddings of all proteins observed in at least 9 samples across the environment. These are available in a separate section, *Protein - UMAP Network.* These embeddings are searchable by range of the graph, as well as by Jaccard and Bray-Curtis similarity to known degrading proteins, in order to easily find proteins which were in the local protein space that was observed to be enriched as described previously. We additionally provide access to an interactive graph of this UMAP space, so researchers may also browse this space directly. Metagenomic abundance information for each gene in the *MetaG_Proteins* is also included, with details on the metadata associated with the samples in which the protein was observed. Metatranscriptomic abundance information for genes from Wu and colleagues is available in the *MetaT_Proteins* section.

We also make available the 90% amino acid identity dereplicated plastisphere protein set searchable by online BLAST or Diamond (via download). Researchers will be able query their own sequences against PMDB to ascertain where a protein has been observed *in-situ,* as well its location in the protein UMAP network space. This will allow scientists to quickly gather detailed information on their sequence of interest, as well as whether a protein has high likelihood of being capable of degrading plastic based on the meta-omic datasets available in the literature.

**Prokaryotic genomes with reported biodegradation of plastic**

The plastic with the most reported degrading prokaryotic genomospecies was polyethylene, followed by poly(lactic acid) (PLA) and poly(ethylene terephthalate) (PET) (Supplementary Figures 1-2). Polyethylene (PE) is largely considered a recalcitrant polymer^1^. However, this plastic had the widest phylogenetic distribution of reported degradation activity of any other plastic. Primarily reported were degraders of low-density polyethylene (LDPE), though there were some reports of degradation of high-density polyethylene (HDPE). There are still very few known enzymes with rapid degradation activity on polyethylene, the most rapidly degrading enzyme being from the recent waxworm study by Sanluis-Verdes and colleages^2^. Homologs to these two enzymes were not seen in any of these 142 available bacterial genomes.

To note, this does not lead us to conclude that polyethylene is more biodegradable than more traditional biopolymers such as polyhydroxybutyrate (PHB) and polycaprolactone (PCL), as the latter likely have had lower isolation efforts due to their known more rapid biodegradability^3^. Biopolymers such as PHB and PCL also generally biodegraded rapidly in the isolation studies we observed. Details on the articles containing plastic associated isolates are listed in Supplemental File 2.

**Supplementary Figure 1. Phylogenetic Tree of Plastic Associated Prokaryotes**. Squares show the plastics reported to be degraded by the microbe. Black boxes represent isolates without confirmed degradation activity, the white boxes beneath these show the plastics from which this microbe was isolated.


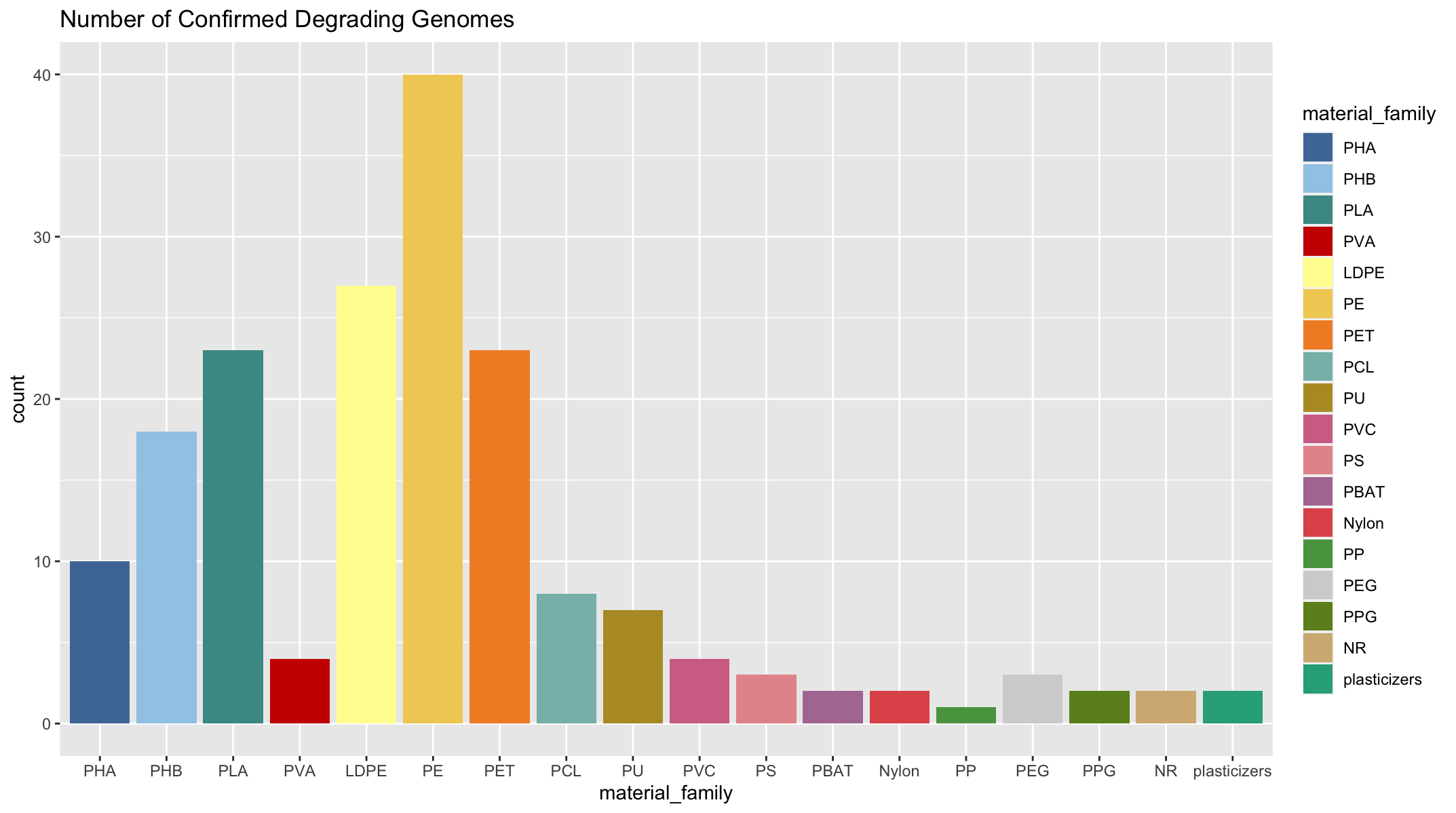


**Supplementary Figure 2.** Number of Confirmed Degrading Genomes per Plastic Type

| **genus** | **rab.all** | **diff.btw** | **diff.win** | **effect** | **wi.ep** | **wi.eBH** | **class** | **order** | **Family** |
| --- | --- | --- | --- | --- | --- | --- | --- | --- | --- |
| g__Henriciella | 2.012 | 4.343 | 5.480 | 0.712 | 8.50E-07 | 3.06E-04 | c__Alphaproteobacteria | o__Caulobacterales | f__Hyphomonadaceae |
| g__JABSSA01 | 0.845 | 3.208 | 5.722 | 0.531 | 2.35E-04 | 9.63E-03 | c__Alphaproteobacteria | o__Rhodobacterales | f__Rhodobacteraceae |
| g__Leptothoe | 0.686 | 3.215 | 6.327 | 0.483 | 3.54E-03 | 4.96E-02 | c__Cyanobacteriia | o__Phormidesmiales | f__Phormidesmiaceae |
| g__Tateyamaria | 0.777 | 2.783 | 6.163 | 0.418 | 9.36E-03 | 9.05E-02 | c__Alphaproteobacteria | o__Rhodobacterales | f__Rhodobacteraceae |
| g__Rivularia | 0.615 | 2.777 | 6.563 | 0.415 | 1.02E-02 | 8.67E-02 | c__Cyanobacteriia | o__Cyanobacteriales | f__Nostocaceae |
| g__unclassified S03_3c9861_ros_idb_n.25_sub | 0.528 | 2.368 | 5.241 | 0.404 | 6.05E-03 | 6.58E-02 | c__Cyanobacteriia | o__PCC-6307 | f__unclassified S03_3c9861_ros_idb_n.25_sub |
| g__Phormidesmis | 0.765 | 2.419 | 6.133 | 0.362 | 1.01E-02 | 9.31E-02 | c__Cyanobacteriia | o__Phormidesmiales | f__Phormidesmiaceae |
| g__MED-G52 | 1.358 | -1.945 | 5.308 | -0.342 | 1.63E-02 | 1.08E-01 | c__Alphaproteobacteria | o__Rhodobacterales | f__Rhodobacteraceae |
| g__UBA9145 | 1.366 | -2.022 | 4.974 | -0.358 | 1.49E-02 | 9.98E-02 | c__Gammaproteobacteria | o__Pseudomonadales | f__Pseudohongiellaceae |
| g__UBA8309 | 1.412 | -2.139 | 5.249 | -0.367 | 1.00E-02 | 8.41E-02 | c__Alphaproteobacteria | o__Puniceispirillales | f__Puniceispirillaceae |
| g__UBA724 | 1.378 | -2.007 | 5.031 | -0.367 | 1.25E-02 | 9.51E-02 | c__Bacteroidia | o__Flavobacteriales | f__Flavobacteriaceae |
| g__R2A130 | 1.562 | -2.179 | 5.281 | -0.371 | 1.11E-02 | 9.49E-02 | c__Alphaproteobacteria | o__Rhizobiales | f__Rhizobiaceae |
| g__Boseongicola | 1.561 | -2.213 | 5.154 | -0.383 | 8.27E-03 | 7.71E-02 | c__Alphaproteobacteria | o__Rhodobacterales | f__Rhodobacteraceae |
| g__JABDJO01 | 1.515 | -2.273 | 5.059 | -0.394 | 6.91E-03 | 7.38E-02 | c__Alphaproteobacteria | o__Rhodobacterales | f__Rhodobacteraceae |
| g__UBA10364 | 1.497 | -2.211 | 5.165 | -0.396 | 7.15E-03 | 6.92E-02 | c__Bacteroidia | o__Flavobacteriales | f__Schleiferiaceae |
| g__UBA1268 | 1.724 | -2.301 | 4.928 | -0.414 | 3.19E-03 | 4.23E-02 | c__Planctomycetia | o__Pirellulales | f__UBA1268 |
| g__Luminiphilus | 1.578 | -2.255 | 4.890 | -0.416 | 5.37E-03 | 5.89E-02 | c__Gammaproteobacteria | o__Pseudomonadales | f__Halieaceae |
| g__UBA11606 | 1.796 | -2.264 | 4.670 | -0.435 | 2.12E-03 | 3.31E-02 | c__Acidimicrobiia | o__Acidimicrobiales | f__UBA11606 |
| g__HIMB30 | 1.706 | -2.497 | 5.127 | -0.436 | 3.09E-03 | 4.05E-02 | c__Gammaproteobacteria | o__Pseudomonadales | f__Litoricolaceae |
| g__Synechococcus_C | 1.859 | -2.779 | 5.679 | -0.457 | 1.10E-03 | 2.37E-02 | c__Cyanobacteriia | o__PCC-6307 | f__Cyanobiaceae |
| g__HIMB11 | 1.952 | -2.673 | 4.840 | -0.484 | 7.00E-04 | 1.71E-02 | c__Alphaproteobacteria | o__Rhodobacterales | f__Rhodobacteraceae |
| g__UBA4421 | 2.234 | -3.089 | 4.988 | -0.554 | 1.14E-04 | 5.49E-03 | c__Gammaproteobacteria | o__Pseudomonadales | f__HTCC2089 |
| g__Synechococcus_E | 2.878 | -4.375 | 5.365 | -0.730 | 3.10E-06 | 5.16E-04 | c__Cyanobacteriia | o__PCC-6307 | f__Cyanobiaceae |

**Supplemental Table 1.** **Genera enriched in degraded PE & PP samples versus all other non-degraded oceanic samples**. Only significantly enriched groups by ALDEx2 analysis (adjusted p-value less than 0.1) are shown. Diff.btw – median centered-log ratio (clr) difference between groups , Diff.win – median of largest difference in clr values within groups, wi.eBH- Expected Benjamini-Hochberg corrected p-value of wilcoxon test.

| **species** | **diff.btw** | **diff.win** | **effect** | **genus** | **wi.eBH** |
| --- | --- | --- | --- | --- | --- |
| s__unclassified S07_18r1486_mtb_spa_t.17 | 2.124 | 5.442 | 0.347 | g__Henriciella | 0.231 |
| s__unclassified S07_18r1486_ros_spa_n.1 | 1.280 | 5.143 | 0.223 | g__Henriciella | 0.387 |
| s__unclassified S18_41ao8643_mtb_idb_t.15 | 0.893 | 4.899 | 0.159 | g__Henriciella | 0.488 |
| s__unclassified S18_44ar8646_mtb_spa_t.12 | 0.739 | 4.745 | 0.138 | g__Henriciella | 0.576 |
| s__unclassified S18_30ad8632_mtb_spa_n.1 | 0.762 | 4.939 | 0.133 | g__Henriciella | 0.580 |
| s__unclassified S07_23w1491_mtb_idb_n.8 | 0.117 | 4.497 | 0.023 | g__Henriciella | 0.767 |
| s__unclassified S18_40an8642_mtb_idb_n.6_sub | 0.001 | 4.537 | 0.000 | g__Henriciella | 0.764 |
| s__unclassified S07_21u1489_mtb_idb_n.17 | -0.018 | 4.595 | -0.003 | g__Henriciella | 0.756 |
| s__unclassified S18_44ar8646_mtb_spa_t.10 | -0.152 | 4.457 | -0.031 | g__Henriciella | 0.750 |
| s__unclassified S03_8h9866_mtb_spa_t.8 | -0.204 | 4.284 | -0.042 | g__Henriciella | 0.746 |
| s__unclassified S22_4d1151_mtb_idb_t.3 | -0.205 | 4.273 | -0.043 | g__Henriciella | 0.729 |
| s__Henriciella sp013213825 | -0.236 | 4.306 | -0.047 | g__Henriciella | 0.734 |
| s__Henriciella algicola | -0.281 | 4.255 | -0.055 | g__Henriciella | 0.702 |
| s__unclassified S09_2b3130_mtb_idb_t.12_sub | -0.308 | 4.306 | -0.064 | g__Henriciella | 0.692 |
| s__unclassified OceanDNA-b23505 | -0.328 | 4.232 | -0.070 | g__Henriciella | 0.718 |
| s__unclassified OceanDNA-b23499 | -0.360 | 4.272 | -0.071 | g__Henriciella | 0.691 |
| s__Henriciella sp002172915 | -0.363 | 4.271 | -0.076 | g__Henriciella | 0.682 |
| s__unclassified OceanDNA-b23503 | -0.580 | 4.349 | -0.119 | g__Henriciella | 0.629 |
| s__unclassified OceanDNA-b23517 | -0.647 | 4.462 | -0.127 | g__Henriciella | 0.623 |
| s__unclassified OceanDNA-b23491 | -0.684 | 4.488 | -0.131 | g__Henriciella | 0.591 |

**Supplemental Table 2**. **Enrichment of *Henricella* genomes in PE & PP plastic vs. control ocean metagenomes.** Only significantly enriched groups *by* ALDEx2 *analysis* (adjusted p-value less than 0.1) are shown. Subset of only genomes from Henriciella genus are shown. Abbreviations are identical to those in Supplemental Table 1.

**KEGG metabolism**

The available plastic samples from wastewater, were primarily composed of polystyrene samples from Li and colleages^4^. These samples did not show as strong enrichment patterns as the riverine samples when compared to control wastewater samples. Instead, wastewater samples only showed the enrichment of a few potentially plastic-compound related pathways such as degradation of glycans and vitamin B6 production. The few wastewater samples for which degradation was reported did show additional enrichment for degradation of PAH and other aromatic pathways, as well as pathways for biofilm formation.

As the available soil samples primarily consisted of more readily degradable plastics (PBAT, PLA, PE), we expected to observe a greater number of KEGG pathways relating to biodegradation of related compounds in this habitat. PAH degradation and biofilm formation pathways were again enriched in plastic degradation associated soil samples. Increased in presence across all plastic samples in the soil vs. controls were also the degradation of steroids, caprolactam, and terpenoids.

We did not observe general enrichment patterns at the KEGG metabolism level for putative degradation pathways in the ocean environment, likely due to the wide variety of controls within this sample set, as well as the inadequate functional annotation present for many genes in this group. Across all other environments in the plastisphere vs. controls, we observed the enrichment in degradation pathways for various polymer related aromatics, such as caprolactam, toluene, and styrene. Additionally enriched were many amino acid production pathways, as well as radical generating pathways such as cytochrome P450. Beta oxidation pathways and synthesis of polysaccharides was also commonly observed as abundant. These results suggest that in the environment, plastic may be utilized similarly to natural polymeric biofilms, selecting for microbes that are able to utilize their chemical components and integrate them into cellular biopolymers. Radical oxidation appears to be a widely used mechanism for utilization of and survival on these recalcitrant compounds. Notably, there is a lack of anaerobic samples within this dataset, thus these findings would obviously apply only to aerobic environments.


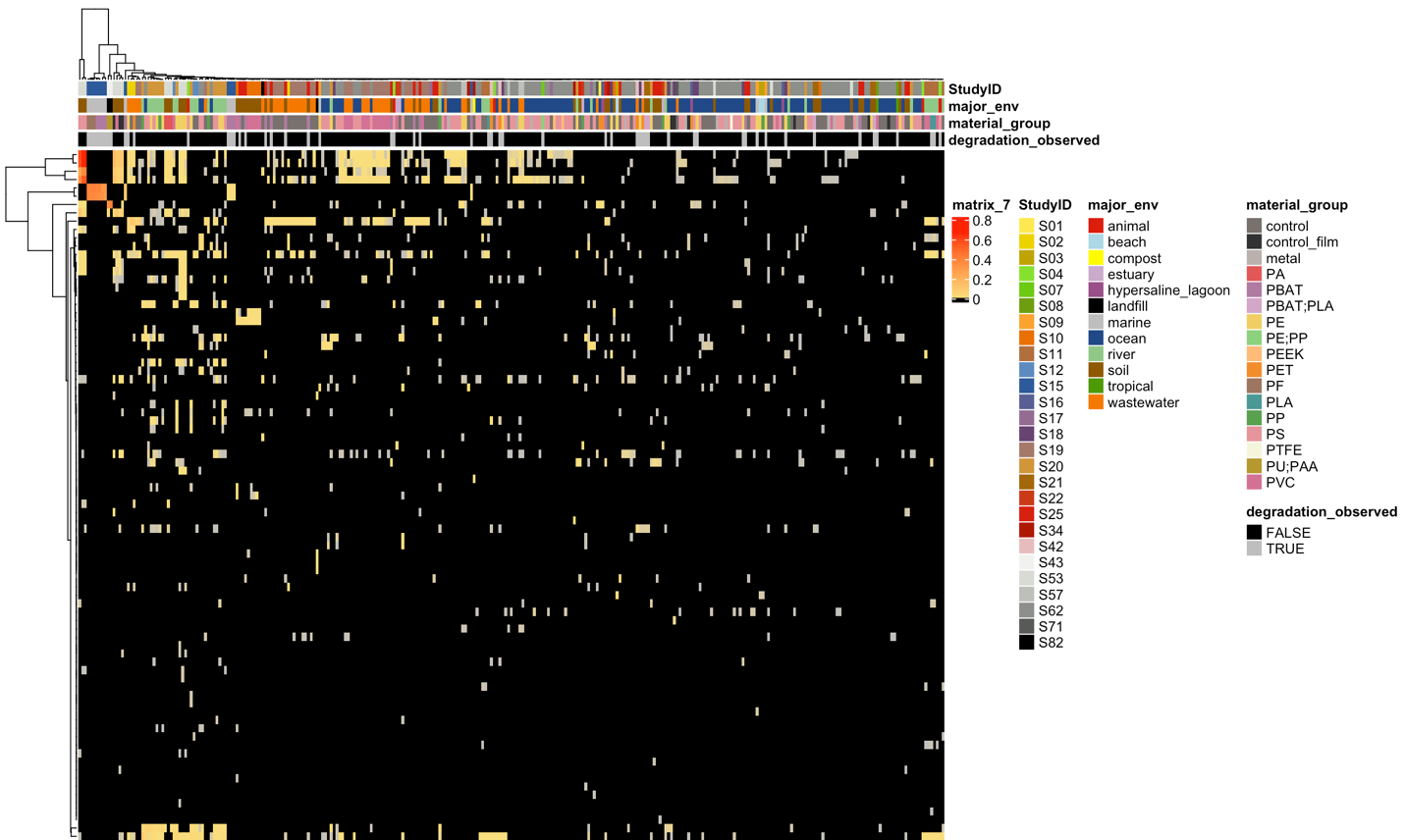


**Supplemental Figure 3.** Distribution of known plastic degrading genes in environmental metagenomes. Rows correspond to known genes, and columns correspond to samples. Gene presence was determined by truncated average depth values of metagenomes (TAD90) mapped to the plastisphere gene set, normalized by genomic equivalents. Only genes known to degrade plastic are shown. Heatmap bars contain metadata relating to the specific sample.


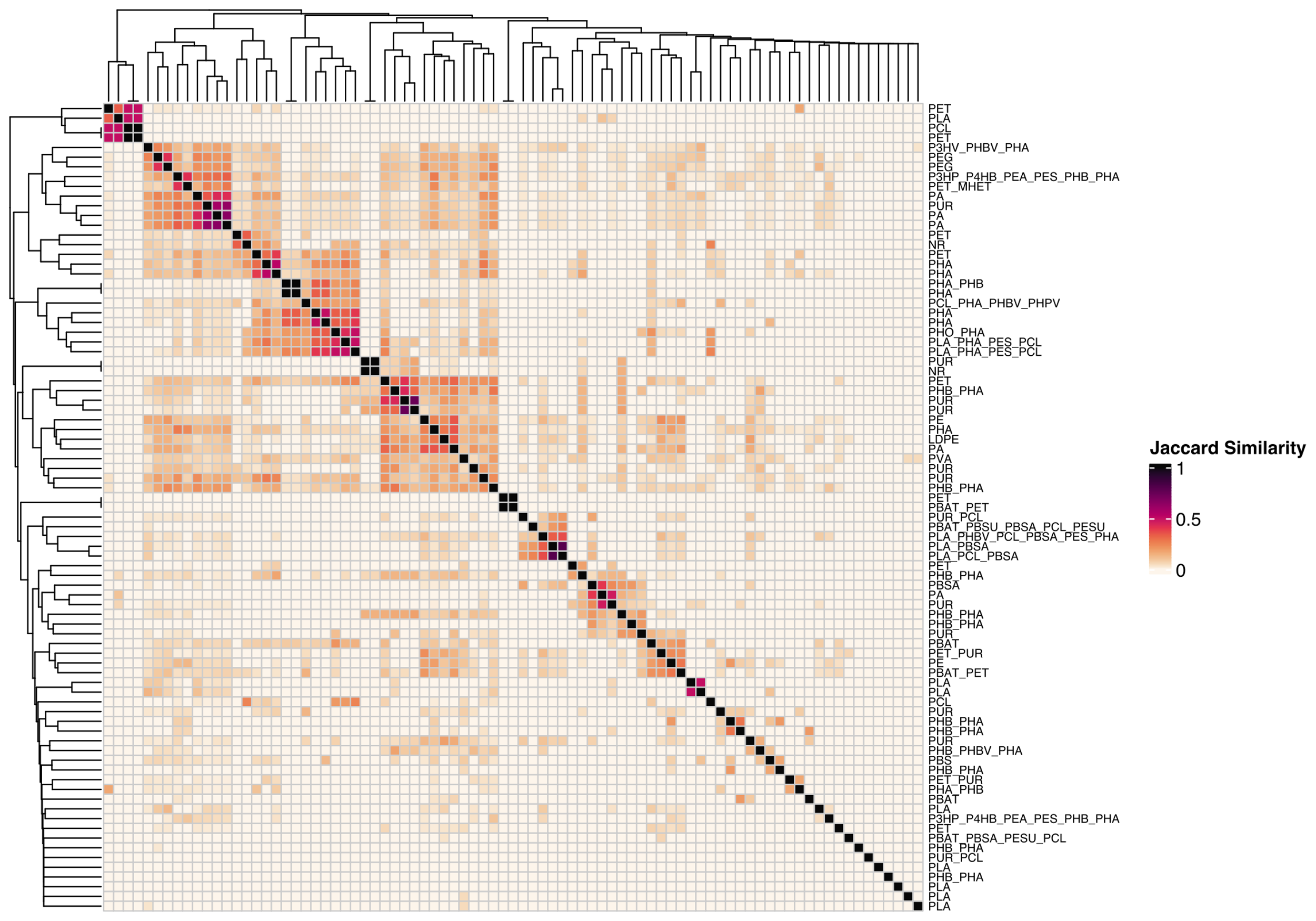


**Supplemental Figure 4.** Jaccard similarity of known plastic degrading genes across environmental metagenomes. Clusters indicate groups of plastic degrading genes which were often seen in the same metagenomic samples across the plastisphere. Row names indicate the types of plastic degraded by the corresponding gene.

**Supplemental Figure 5.** UMAP Network Graph colored by enrichment in metatranscriptomic dataset from Wu and colleagues. Orange dots are specific locations of proteins known to degrade plastic. Colors are pseudo-log fold change (difference between) in polyvinyl chloride (PVC) versus polylactic acid (PLA) samples calculated via ALDEx2.

**
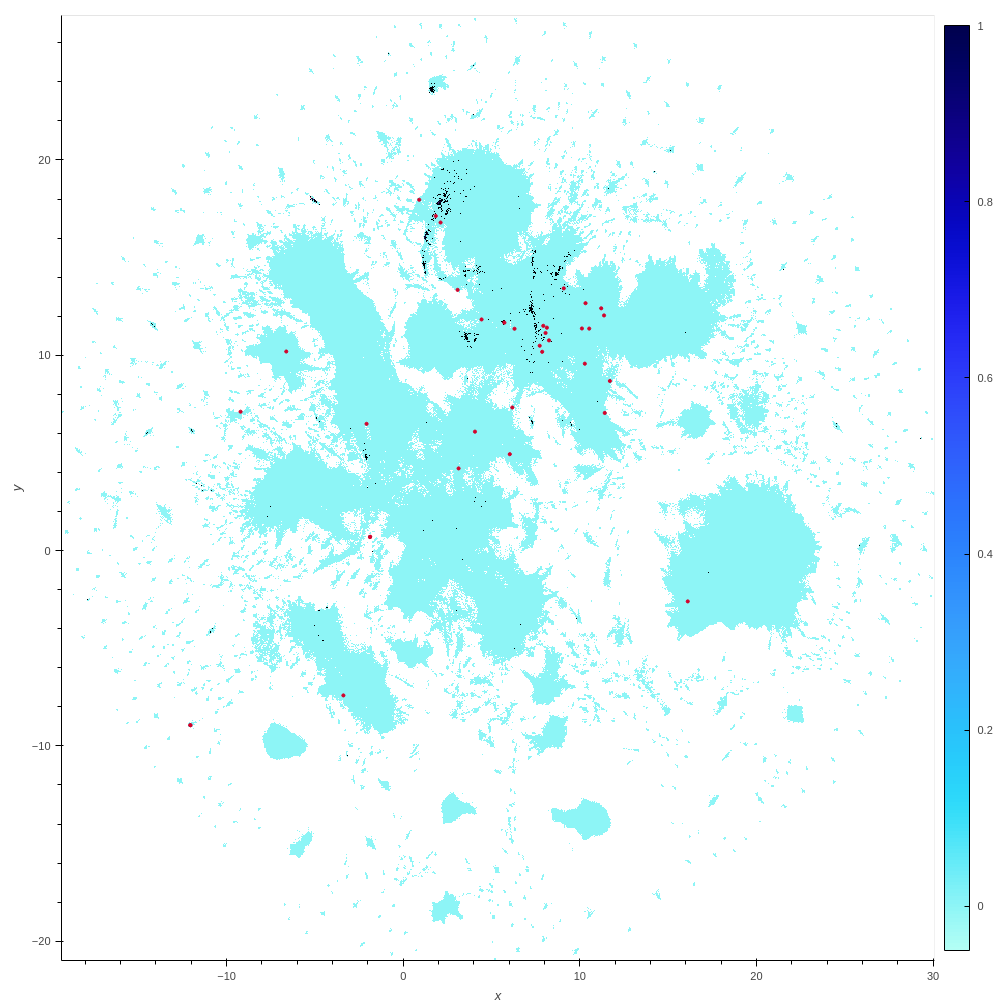
**

**Supplemental Figure 6 - UMAP Network Graph showing location of genes from other ‘omics studies.** Red dots are specific locations of proteins known to degrade plastic. Black dots are the locations of genes which were observed in transcriptomic, proteomic, or metaproteomic datasets. Of note is the dense regions of black points within the same region as many genes known to perform plastic biodegradation. Metatranscriptomic genes from Wu and colleagues (Supplemental Figure 5) are not specifically selected in this plot.

#

***Supplemental Figure 7 – Snakemake Pipeline for Paired-End Metagenome Reconstruction and Analysis*.** Pipeline shows the start to end workflow developed in snakemake for the analysis of metagenomic data. Further details on the workflow may be found in the Materials and Methods section.

***Supplemental Figure 8 – 16S prevalence of known plastic degraders in riverine samples*.** Plot shows relative abundance of species known to degrade plastics across various conditions in riverine systems. The top row, ‘Non-Plastic’ refers to 16S samples which did not contain plastic, collected either from water or alternative biofilms. The bottom refers to samples from plastic biofilms. The left column (Environmental Sample) is samples from direct environmental incubations, while the right column is mesocosm incubations designed to replicate riverine conditions. The clear enrichment of plastic degrading species is observed in plastic samples which have undergone direct environmental incubations. The underlying data is 16S amplicon assigned to a known plastic degrader isolate (y-axes) based on a >98.5% nucleotide identity match to the isolate 16S sequence. Sequence accessions and metadata are contained in Supplemental File 5.

**Effects of Alpha Diversity**

#
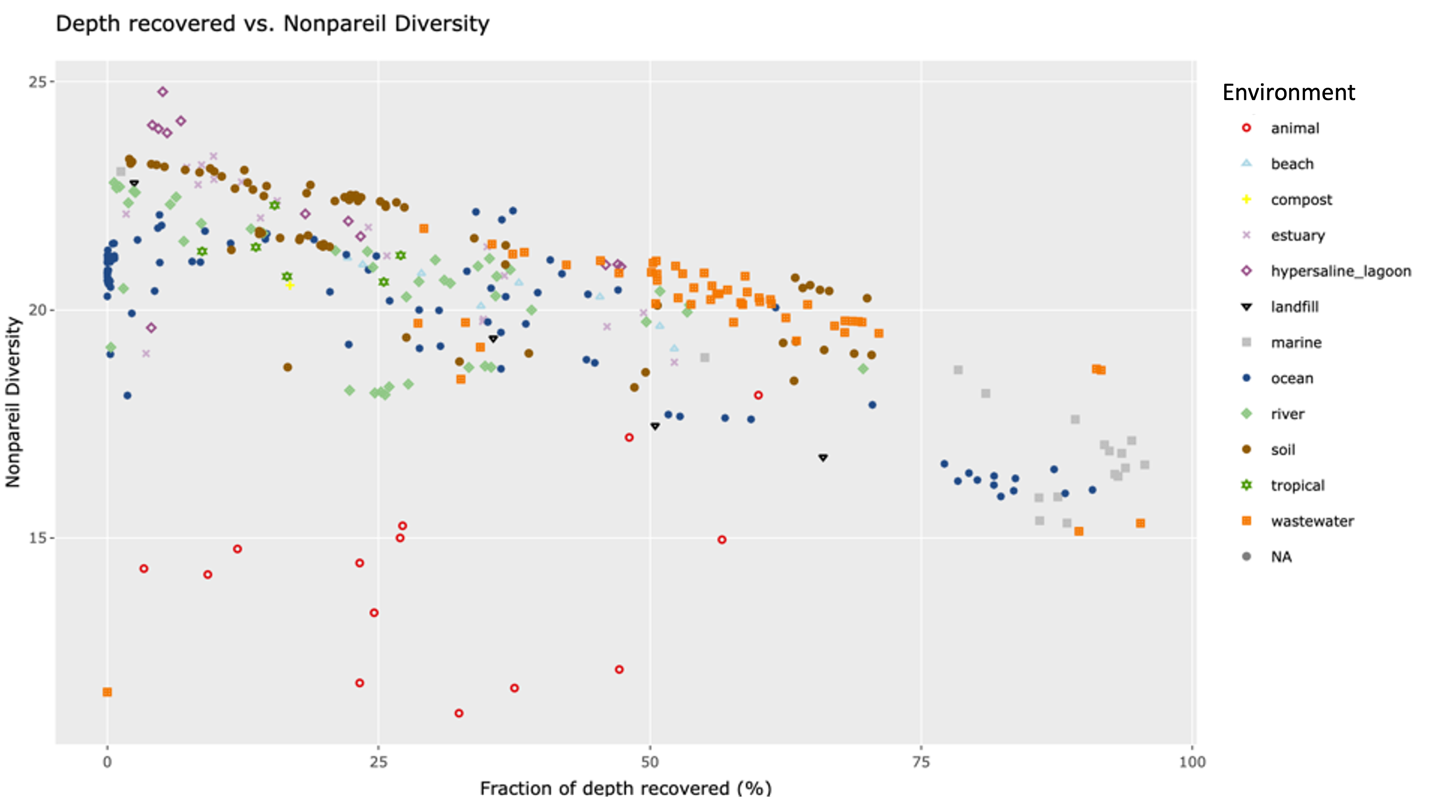


# ***Supplemental Figure 9 – Estimated Nonpareil diversity vs. the fraction of depth recovered.*** *Nonpareil diversity corresponds to the estimated alpha-diversity of a given sample. The ‘fraction of depth recovered’ is the estimated percentage of this diversity which was sampled in the corresponding metagenome. Legend shows the symbols and colors used for samples from various environments.*

Within this study, there is a variety of environment types, in addition to the added variance in sequencing effort and number of samples available for each environment. Thus, a consideration of the effects of within-sample diversity is useful. We therefore performed an alpha-diversity like metric for the samples of this study using Nonpareil^5^, as noted in the Materials and Methods section. Nonpareil diversity corresponds to the estimated alpha-diversity of a given sample. This analysis included all metagenomic samples which were from short reads (Nonpareil cannot provide estimates for long reads), which is the vast majority of all samples included in the study. Only 4 long read samples are not present, none of which displayed significant known plastic degrading populations.

# Within this data, we observe the expected pattern of lower fractions of the total diversity sampled for metagenomes with a higher estimated diversity, as seen in Supp. Fig. 9. Predictably, we find many of our soil samples have a relatively high diversity and thus lower fraction of depth recovered. We do also observe estuarine, lagoon, and marine samples with similarly high levels of diversity.

# This study contains more samples of both soil (n=71) and wastewater (n=53) environments than that of the river (n=42); these environments each follow the same overall trend of reduced fraction of depth recovered with increasing diversity. Notably, however, the wastewater samples (orange squares) generally have a higher fraction of their diversity sampled than riverine samples (green diamonds). We also have soil samples (brown circles) with both higher and lower diversity estimates than the riverine samples. Thus, these soil and wastewater samples enclose both sides of diversity and fractions recovered, and thus also would likely capture the corresponding potential variance. We therefore do not expect that the alpha diversity is the cause of the enrichment or lack thereof of plastic degrading microbes within the observed populations. **REFERENCES**

1. Ghatge, S., Yang, Y., Ahn, J.-H. & Hur, H.-G. Biodegradation of polyethylene: a brief review. *Appl. Biol. Chem.* **63**, 27 (2020).

2. Sanluis-Verdes, A. *et al.* Wax worm saliva and the enzymes therein are the key to polyethylene degradation by Galleria mellonella. *Nat. Commun.* **13**, 5568 (2022).

3. Suzuki, M., Tachibana, Y. & Kasuya, K. Biodegradability of poly(3-hydroxyalkanoate) and poly(ε-caprolactone) via biological carbon cycles in marine environments. *Polym. J.* **53**, 47–66 (2021).

4. Li, Q., Tian, L., Cai, X., Wang, Y. & Mao, Y. Plastisphere showing unique microbiome and resistome different from activated sludge. *Sci. Total Environ.* **851**, 158330 (2022).

5. Rodriguez-R, L. M., Gunturu, S., Tiedje, J. M., Cole, J. R. & Konstantinidis, K. T. Nonpareil 3: Fast Estimation of Metagenomic Coverage and Sequence Diversity. *mSystems* **3**, (2018).

6. Muhonja, C. N., Makonde, H., Magoma, G. & Imbuga, M. Biodegradability of polyethylene by bacteria and fungi from Dandora dumpsite Nairobi-Kenya. *PLOS ONE* **13**, e0198446 (2018).

7. Montazer, Z., Habibi Najafi, M. B. & Levin, D. B. Microbial degradation of low-density polyethylene and synthesis of polyhydroxyalkanoate polymers. *Can. J. Microbiol.* **65**, 224–234 (2019).

8. Schneider, B., Pfeiffer, F., Dyall-Smith, M. & Kunte, H.-J. Genome Sequence of Micromonospora aurantiaca Strain G9, a Member of a Bacterial Consortium Capable of Polyethylene Degradation. *Microbiol. Resour. Announc.* **11**, e01148-21 (2022).

9. Schneider, B., Pfeiffer, F., Dyall-Smith, M. & Kunte, H.-J. Genome Sequence of Pseudomonas veronii Strain G2, a Member of a Bacterial Consortium Capable of Polyethylene Degradation. *Microbiol. Resour. Announc.* **11**, e00365-22 (2022).

10. Schneider, B., Pfeiffer, F., Dyall-Smith, M. & Kunte, H.-J. Genome Sequence of Cupriavidus campinensis Strain G5, a Member of a Bacterial Consortium Capable of Polyethylene Degradation. *Microbiol. Resour. Announc.* **11**, e00553-22 (2022).

11. Roy, R., Mukherjee, G., Das Gupta, A., Tribedi, P. & Sil, A. K. Isolation of a soil bacterium for remediation of polyurethane and low-density polyethylene: a promising tool towards sustainable cleanup of the environment. *3 Biotech* **11**, 29 (2021).

12. Lee, B., Pometto, A. L., Fratzke, A. & Bailey, T. B. Biodegradation of Degradable Plastic Polyethylene by Phanerochaete and Streptomyces Species. *Appl. Environ. Microbiol.* **57**, 678–685 (1991).

13. Lear, L. *et al.* Bacterial colonisation dynamics of household plastics in a coastal environment. *Sci. Total Environ.* **838**, 156199 (2022).

14. Kasai, D. *et al.* Identification of natural rubber degradation gene in Rhizobacter gummiphilus NS21. *Biosci. Biotechnol. Biochem.* **81**, 614–620 (2017).

15. Tsuchii, A. & Takeda, K. Rubber-Degrading Enzyme from a Bacterial Culture. *Appl. Environ. Microbiol.* **56**, 269–274 (1990).

16. Kinoshita, S., Kageyama, S., Iba, K., Yamada, Y. & Okada, H. Utilization of a Cyclic Dimer and Linear Oligomers of ε-Aminocaproic Acid by Achrornobacter guttatus KI 72. *Agric. Biol. Chem.* **39**, 1219–1223 (1975).

17. Park, G.-S. *et al.* Complete genome sequence of the caprolactam-degrading bacterium Pseudomonas mosselii SJ10 isolated from wastewater of a nylon 6 production plant. *J. Biotechnol.* **192**, 263–264 (2014).

18. Klomklang, W. *et al.* Biochemical and molecular characterization of a periplasmic hydrolase for oxidized polyvinyl alcohol from Sphingomonas sp. strain 113P3. *Microbiology* **151**, 1255–1262 (2005).

19. Soulenthone, P. *et al.* Characterization of a poly(butylene adipate-co-terephthalate) hydrolase from the mesophilic actinobacteria Rhodococcus fascians. *Polym. Degrad. Stab.* **184**, 109481 (2021).

20. Muroi, F. *et al.* Characterization of a poly(butylene adipate- co -terephthalate) hydrolase from the aerobic mesophilic bacterium Bacillus pumilus. *Polym. Degrad. Stab.* **137**, 11–22 (2017).

21. Danso, D. *et al.* New Insights into the Function and Global Distribution of Polyethylene Terephthalate (PET)-Degrading Bacteria and Enzymes in Marine and Terrestrial Metagenomes. *Appl. Environ. Microbiol.* **84**, e02773-17 (2018).

22. Lee, S.-H. *et al.* Complete genome of biodegradable plastics-decomposing Roseateles depolymerans KCTC 42856T (=61AT). *J. Biotechnol.* **220**, 47–48 (2016).

23. Molitor, R. *et al.* Agar plate‐based screening methods for the identification of polyester hydrolysis by Pseudomonas species. *Microb. Biotechnol.* **13**, 274–284 (2020).

24. Freitas, R. C. D., Odisi, E. J., Kato, C., Da Silva, M. A. C. & Lima, A. O. D. S. Draft Genome Sequence of the Deep-Sea Bacterium Moritella sp. JT01 and Identification of Biotechnologically Relevant Genes. *Mar. Biotechnol.* **19**, 480–487 (2017).

25. Yang, J., Yang, Y., Wu, W.-M., Zhao, J. & Jiang, L. Evidence of Polyethylene Biodegradation by Bacterial Strains from the Guts of Plastic-Eating Waxworms. *Environ. Sci. Technol.* **48**, 13776–13784 (2014).

26. Zhang, J. *et al.* Selection and evaluation of microorganisms for biodegradation of agricultural plastic film. *3 Biotech* **8**, 308 (2018).

27. Shao, H. *et al.* Complete Genome Sequence and Characterization of a Polyethylene Biodegradation Strain, Streptomyces Albogriseolus LBX-2. *Microorganisms* **7**, 379 (2019).

28. Peixoto, J. *et al.* The role of nitrogen metabolism on polyethylene biodegradation. *J. Hazard. Mater.* **432**, 128682 (2022).

29. Furlan, J. P. R., Lopes, R. & Stehling, E. G. Whole-genome sequence-based analysis of the Paenibacillus aquistagni strain DK1, a polyethylene-degrading bacterium isolated from landfill. *World J. Microbiol. Biotechnol.* **37**, 80 (2021).

30. Liu, X. *et al.* Rapid colonization and biodegradation of untreated commercial polyethylene wrap by a new strain of Bacillus velezensis C5. *J. Environ. Manage.* **301**, 113848 (2022).

31. Hou, L. *et al.* Biodegradability of polyethylene mulching film by two Pseudomonas bacteria and their potential degradation mechanism. *Chemosphere* **286**, 131758 (2022).

32. Borre, I. & Sonnenschein, E. C. Draft Genome Sequences of Nine Environmental Bacterial Isolates Colonizing Plastic. *Microbiol. Resour. Announc.* **10**, e01485-20 (2021).

33. Calabia, B. P. & Tokiwa, Y. A Novel PHB Depolymerase from a Thermophilic Streptomyces Sp. *Biotechnol. Lett.* **28**, 383–388 (2006).

34. Austin, H. P. *et al.* Characterization and engineering of a plastic-degrading aromatic polyesterase. *Proc. Natl. Acad. Sci.* **115**, (2018).

35. Yamashita, M., Tani, A. & Kawai, F. A new ether bond-splitting enzyme found in Gram-positive polyethylene glycol 6000-utilizing bacterium, Pseudonocardia sp. strain K1. *Appl. Microbiol. Biotechnol.* **66**, 174–179 (2004).

36. Ohtsubo, Y. *et al.* Complete Genome Sequence of Sphingopyxis macrogoltabida Strain 203N (NBRC 111659), a Polyethylene Glycol Degrader. *Genome Announc.* **4**, e00529-16 (2016).

37. Chertkov, O. *et al.* Complete genome sequence of Thermomonospora curvata type strain (B9T). *Stand. Genomic Sci.* **4**, 13–22 (2011).

38. Zampolli, J. *et al.* Transcriptomic analysis of Rhodococcus opacus R7 grown on polyethylene by RNA-seq. *Sci. Rep.* **11**, 21311 (2021).

39. Herrero Acero, E. *et al.* Enzymatic Surface Hydrolysis of PET: Effect of Structural Diversity on Kinetic Properties of Cutinases from Thermobifida. *Macromolecules* **44**, 4632–4640 (2011).

40. Bollinger, A. *et al.* A Novel Polyester Hydrolase From the Marine Bacterium Pseudomonas aestusnigri – Structural and Functional Insights. *Front. Microbiol.* **11**, 114 (2020).

41. Wright, R. J., Bosch, R., Gibson, M. I. & Christie-Oleza, J. A. Plasticizer Degradation by Marine Bacterial Isolates: A Proteogenomic and Metabolomic Characterization. *Environ. Sci. Technol.* **54**, 2244–2256 (2020).

42. Wright, R. J., Bosch, R., Langille, M. G. I., Gibson, M. I. & Christie-Oleza, J. A. A multi-OMIC characterisation of biodegradation and microbial community succession within the PET plastisphere. *Microbiome* **9**, 141 (2021).

43. Haernvall, K. *et al.* Hydrolysis of Ionic Phthalic Acid Based Polyesters by Wastewater Microorganisms and Their Enzymes. *Environ. Sci. Technol.* **51**, 4596–4605 (2017).

44. León-Zayas, R., Roberts, C., Vague, M. & Mellies, J. L. Draft Genome Sequences of Five Environmental Bacterial Isolates That Degrade Polyethylene Terephthalate Plastic. *Microbiol. Resour. Announc.* **8**, e00237-19 (2019).

45. Liu, R., Lai, Q., Gu, L., Yan, P. & Shao, Z. Croceimicrobium hydrocarbonivorans gen. nov., sp. nov., a novel marine bacterium isolated from a bacterial consortium that degrades polyethylene terephthalate. *Int. J. Syst. Evol. Microbiol.* **71**, (2021).

46. Gao, R. & Sun, C. A marine bacterial community capable of degrading poly(ethylene terephthalate) and polyethylene. *J. Hazard. Mater.* **416**, 125928 (2021).

47. Lago-Maciel, A., Nielsen, T. K., Jensen, K., Nicolaisen, M. H. & Hennessy, R. C. Complete Genome Sequence of Sphingopyxis sp. Strain PET50, a Potential Polyethylene Terephthalate (PET)-Degrading Bacterium Isolated from Compost. *Microbiol. Resour. Announc.* **12**, e00970-22 (2023).

48. De Eugenio, L. I. *et al.* Biochemical Evidence That phaZ Gene Encodes a Specific Intracellular Medium Chain Length Polyhydroxyalkanoate Depolymerase in Pseudomonas putida KT2442. *J. Biol. Chem.* **282**, 4951–4962 (2007).

49. Martínez, V. *et al.* Identification and Biochemical Evidence of a Medium-Chain-Length Polyhydroxyalkanoate Depolymerase in the Bdellovibrio bacteriovorus Predatory Hydrolytic Arsenal. *Appl. Environ. Microbiol.* **78**, 6017–6026 (2012).

50. De Vogel, F. A., Schlundt, C., Stote, R. E., Ratto, J. A. & Amaral-Zettler, L. A. Comparative Genomics of Marine Bacteria from a Historically Defined Plastic Biodegradation Consortium with the Capacity to Biodegrade Polyhydroxyalkanoates. *Microorganisms* **9**, 186 (2021).

51. Morohoshi, T., Oi, T., Suzuki, T. & Sato, S. Identification and characterization of a novel extracellular polyhydroxyalkanoate depolymerase in the complete genome sequence of Undibacterium sp. KW1 and YM2 strains. *PLOS ONE* **15**, e0232698 (2020).

52. Sznajder, A. & Jendrossek, D. Biochemical characterization of a new type of intracellular PHB depolymerase from Rhodospirillum rubrum with high hydrolytic activity on native PHB granules. *Appl. Microbiol. Biotechnol.* **89**, 1487–1495 (2011).

53. Wang, Y.-L., Lin, Y.-T., Chen, C.-L., Shaw, G.-C. & Liaw, S.-H. Crystallization and preliminary crystallographic analysis of poly(3-hydroxybutyrate) depolymerase from Bacillus thuringiensis. *Acta Crystallogr. Sect. F Struct. Biol. Commun.* **70**, 1421–1423 (2014).

54. Lu, J., Takahashi, A. & Ueda, S. 3-Hydroxybutyrate Oligomer Hydrolase and 3-Hydroxybutyrate Dehydrogenase Participate in Intracellular Polyhydroxybutyrate and Polyhydroxyvalerate Degradation in Paracoccus denitrificans. *Appl. Environ. Microbiol.* **80**, 986–993 (2014).

55. Adaya, L. *et al.* Inactivation of an intracellular poly-3-hydroxybutyrate depolymerase of Azotobacter vinelandii allows to obtain a polymer of uniform high molecular mass. *Appl. Microbiol. Biotechnol.* **102**, 2693–2707 (2018).

56. Tabrez Khan, S. & Hiraishi, A. Diaphorobacter nitroreducens gen. nov., sp. nov., a poly(3-hydroxybutyrate)-degrading denitrifying bacterium isolated from activated sludge. *J. Gen. Appl. Microbiol.* **48**, 299–308 (2002).

57. Martínez-Tobón, D. I., Gul, M., Elias, A. L. & Sauvageau, D. Polyhydroxybutyrate (PHB) biodegradation using bacterial strains with demonstrated and predicted PHB depolymerase activity. *Appl. Microbiol. Biotechnol.* **102**, 8049–8067 (2018).

58. Abe, T., Kobayashi, T. & Saito, T. Properties of a Novel Intracellular Poly(3-Hydroxybutyrate) Depolymerase with High Specific Activity (PhaZd) in Wautersia eutropha H16. *J. Bacteriol.* **187**, 6982–6990 (2005).

59. Elbanna, K., L�tke-Eversloh, T., Jendrossek, D., Luftmann, H. & Steinb�chel, A. Studies on the biodegradability of polythioester copolymers and homopolymers by polyhydroxyalkanoate (PHA)-degrading bacteria and PHA depolymerases. *Arch. Microbiol.* **182**, (2004).

60. Suzuki, M. *et al.* A novel poly(3-hydroxybutyrate)-degrading actinobacterium that was isolated from plastisphere formed on marine plastic debris. *Polym. Degrad. Stab.* **183**, 109461 (2021).

61. Jendrossek, D. *et al.* Biochemical and molecular characterization of the Pseudomonas lemoignei polyhydroxyalkanoate depolymerase system. *J. Bacteriol.* **177**, 596–607 (1995).

62. York, G. M. *et al.* Ralstonia eutropha H16 Encodes Two and Possibly Three Intracellular Poly[ -(−)-3-Hydroxybutyrate] Depolymerase Genes. *J. Bacteriol.* **185**, 3788–3794 (2003).

63. Takeda, M., Kamagata, Y., Ghiorse, W. C., Hanada, S. & Koizumi, J. Caldimonas manganoxidans gen. nov., sp. nov., a poly(3-hydroxybutyrate)-degrading, manganese-oxidizing thermophile. *Int. J. Syst. Evol. Microbiol.* **52**, 895–900 (2002).

64. Suzuki, M. *et al.* Difference in environmental degradability between poly(ethylene succinate) and poly(3-hydroxybutyrate). *J. Polym. Res.* **24**, 217 (2017).

65. Hajighasemi, M. *et al.* Biochemical and Structural Insights into Enzymatic Depolymerization of Polylactic Acid and Other Polyesters by Microbial Carboxylesterases. *Biomacromolecules* **17**, 2027–2039 (2016).

66. Jarerat, A., Pranamuda, H. & Tokiwa, Y. Poly(L‐lactide)‐Degrading Activity in Various Actinomycetes. *Macromol. Biosci.* **2**, 420–428 (2002).

67. Penkhrue, W. *et al.* Effective enhancement of polylactic acid-degrading enzyme production by Amycolatopsis sp. strain SCM_MK2-4 using statistical and one-factor-at-a-time approaches. *Prep. Biochem. Biotechnol.* **47**, 730–738 (2017).

68. Jarerat, A. & Tokiwa, Y. Poly(L-lactide) degradation by Saccharothrix waywayandensis. *Biotechnol. Lett.* **25**, 401–404 (2003).

69. Liang, T.-W., Jen, S.-N., Nguyen, A. & Wang, S.-L. Application of Chitinous Materials in Production and Purification of a Poly(l-lactic acid) Depolymerase from Pseudomonas tamsuii TKU015. *Polymers* **8**, 98 (2016).

70. McCarthy, A. J. & Williams, S. T. Actinomycetes as agents of biodegradation in the environment — a review. *Gene* **115**, 189–192 (1992).

71. Bordel, S., Martín-González, D., Muñoz, R. & Santos-Beneit, F. Genome sequence analysis and characterization of Bacillus altitudinis B12, a polylactic acid- and keratin-degrading bacterium. *Mol. Genet. Genomics* **298**, 389–398 (2023).

72. Ohtsubo, Y. *et al.* Complete Genome Sequence of a Polypropylene Glycol-Degrading Strain, Microbacterium sp. No. 7. *Genome Announc.* **3**, e01400-15 (2015).

73. Kyaw, B. M., Champakalakshmi, R., Sakharkar, M. K., Lim, C. S. & Sakharkar, K. R. Biodegradation of Low Density Polythene (LDPE) by Pseudomonas Species. *Indian J. Microbiol.* **52**, 411–419 (2012).

74. Chuang, S. K., Vrla, G. D., Fröhlich, K. S. & Gitai, Z. Surface association sensitizes Pseudomonas aeruginosa to quorum sensing. *Nat. Commun.* **10**, 4118 (2019).

75. Kim, H.-W. *et al.* Biodegradation of polystyrene by bacteria from the soil in common environments. *J. Hazard. Mater.* **416**, 126239 (2021).

76. Parthasarathy, A. *et al.* Polystyrene Degradation by Exiguobacterium sp. RIT 594: Preliminary Evidence for a Pathway Containing an Atypical Oxygenase. *Microorganisms* **10**, 1619 (2022).

77. Ganesh Kumar, A., Hinduja, M., Sujitha, K., Nivedha Rajan, N. & Dharani, G. Biodegradation of polystyrene by deep-sea Bacillus paralicheniformis G1 and genome analysis. *Sci. Total Environ.* **774**, 145002 (2021).

78. Kallscheuer, N. *et al.* Three novel Rubripirellula species isolated from plastic particles submerged in the Baltic Sea and the estuary of the river Warnow in northern Germany. *Antonie Van Leeuwenhoek* **113**, 1767–1778 (2020).

79. Hung, C.-S. *et al.* Carbon Catabolite Repression and Impranil Polyurethane Degradation in Pseudomonas protegens Strain Pf-5. *Appl. Environ. Microbiol.* **82**, 6080–6090 (2016).

80. Stamps, B. W. *et al.* Finished Genome Sequence of a Polyurethane-Degrading Pseudomonas Isolate. *Genome Announc.* **6**, e00084-18 (2018).

81. Espinosa, M. J. C. *et al.* Toward Biorecycling: Isolation of a Soil Bacterium That Grows on a Polyurethane Oligomer and Monomer. *Front. Microbiol.* **11**, 404 (2020).

82. Wei, Y. *et al.* Bioinformatics Analysis and Characterization of Highly Efficient Polyvinyl Alcohol (PVA)-Degrading Enzymes from the Novel PVA Degrader Stenotrophomonas rhizophila QL-P4. *Appl. Environ. Microbiol.* **84**, e01898-17 (2018).

83. Nogi, Y., Yoshizumi, M. & Miyazaki, M. Thalassospira povalilytica sp. nov., a polyvinyl-alcohol-degrading marine bacterium. *Int. J. Syst. Evol. Microbiol.* **64**, 1149–1153 (2014).

84. Giacomucci, L., Raddadi, N., Soccio, M., Lotti, N. & Fava, F. Polyvinyl chloride biodegradation by Pseudomonas citronellolis and Bacillus flexus. *New Biotechnol.* **52**, 35–41 (2019).

85. Zhang, Z. *et al.* Polyvinyl chloride degradation by a bacterium isolated from the gut of insect larvae. *Nat. Commun.* **13**, 5360 (2022).

86. Gravouil, K. *et al.* Transcriptomics and Lipidomics of the Environmental Strain Rhodococcus ruber Point out Consumption Pathways and Potential Metabolic Bottlenecks for Polyethylene Degradation. *Environ. Sci. Technol.* **51**, 5172–5181 (2017).

87. Zadjelovic, V., Gibson, M. I., Dorador, C. & Christie-Oleza, J. A. Genome of Alcanivorax sp. 24: A hydrocarbon degrading bacterium isolated from marine plastic debris. *Mar. Genomics* **49**, 100686 (2020).

88. Kumari, A., Bano, N., Chaudhary, D. R. & Jha, B. Draft genome sequence of plastic degrading Bacillus sp. AIIW2 isolated from the Arabian ocean. *J. Basic Microbiol.* **61**, 37–44 (2021).

89. Huang, Z., Hong, Q., Lai, Q. & Zhang, Q. Portibacter marinus sp. nov., isolated from the sediment on the surface of plastics and proposal of a novel genus Neolewinella gen. nov. based on the genome-based phylogeny of the family Lewinellaceae. *Int. J. Syst. Evol. Microbiol.* **72**, (2022).

90. Satti, S. M., Castro-Aguirre, E., Shah, A. A., Marsh, T. L. & Auras, R. Genome Annotation of Poly(lactic acid) Degrading Pseudomonas aeruginosa, Sphingobacterium sp. and Geobacillus sp. *Int. J. Mol. Sci.* **22**, 7385 (2021).

91. Ohtsubo, Y. *et al.* Complete Genome Sequence of Polypropylene Glycol- and Polyethylene Glycol-Degrading Sphingopyxis macrogoltabida Strain EY-1. *Genome Announc.* **3**, e01399-15 (2015).

92. Wang, X. *et al.* Complete genome sequence of marine Bacillus sp. Y-01, isolated from the plastics contamination in the Yellow Sea. *Mar. Genomics* **43**, 72–74 (2019).

93. Dimarogona, M. *et al.* Structural and functional studies of a Fusarium oxysporum cutinase with polyethylene terephthalate modification potential. *Biochim. Biophys. Acta BBA - Gen. Subj.* **1850**, 2308–2317 (2015).

94. Deguchi, T., Kakezawa, M. & Nishida, T. Nylon biodegradation by lignin-degrading fungi. *Appl. Environ. Microbiol.* **63**, 329–331 (1997).

95. Navarro, D. *et al.* Large-scale phenotyping of 1,000 fungal strains for the degradation of non-natural, industrial compounds. *Commun. Biol.* **4**, 871 (2021).

96. Shinozaki, Y. *et al.* Biodegradable plastic-degrading enzyme from Pseudozyma antarctica: cloning, sequencing, and characterization. *Appl. Microbiol. Biotechnol.* **97**, 2951–2959 (2013).

97. Vázquez-Alcántara, L., Oliart-Ros, R. M., García-Bórquez, A. & Peña-Montes, C. Expression of a Cutinase of Moniliophthora roreri with Polyester and PET-Plastic Residues Degradation Activity. *Microbiol. Spectr.* **9**, e00976-21 (2021).

98. Koitabashi, M. *et al.* Degradation of biodegradable plastic mulch films in soil environment by phylloplane fungi isolated from gramineous plants. *AMB Express* **2**, 40 (2012).

99. Saeed, S., Iqbal, A. & Deeba, F. Biodegradation study of Polyethylene and PVC using naturally occurring plastic degrading microbes. *Arch. Microbiol.* **204**, 497 (2022).

100. Radwan, O. & Ruiz, O. N. Black Yeast Genomes Assembled from Plastic Fabric Metagenomes Reveal an Abundance of Hydrocarbon Degradation Genes. *Microbiol. Resour. Announc.* **10**, e01459-20 (2021).

101. Paço, A. *et al.* Biodegradation of polyethylene microplastics by the marine fungus Zalerion maritimum. *Sci. Total Environ.* **586**, 10–15 (2017).

102. Yang, S., Liu, M., Long, L., Zhang, R. & Ding, S. Characterization of a cutinase from Myceliophthora thermophila and its application in polyester hydrolysis and deinking process. *Process Biochem.* **66**, 106–112 (2018).

103. Janssen, P. J. *et al.* The Complete Genome Sequence of Cupriavidus metallidurans Strain CH34, a Master Survivalist in Harsh and Anthropogenic Environments. *PLoS ONE* **5**, e10433 (2010).

104. Liu, Z. *et al.* Structural and Functional Studies of Aspergillus oryzae Cutinase: Enhanced Thermostability and Hydrolytic Activity of Synthetic Ester and Polyester Degradation. *J. Am. Chem. Soc.* **131**, 15711–15716 (2009).

105. Wei, R. *et al.* Functional characterization and structural modeling of synthetic polyester-degrading hydrolases from Thermomonospora curvata. *AMB Express* **4**, 44 (2014).

106. Calabia, B. P. & Tokiwa, Y. Microbial degradation of poly(d-3-hydroxybutyrate) by a new thermophilic Streptomyces isolate. *Biotechnol. Lett.* **26**, 15–19 (2004).

107. Tripathy, S. *et al.* Draft Genome Sequence of *Brevibacillus borstelensis* cifa_chp40, a Thermophilic Strain Having Biotechnological Importance. *J. Genomics* **4**, 4–6 (2016).

108. Jones, C. J., Grotewold, N., Wozniak, D. J. & Gloag, E. S. Pseudomonas aeruginosa Initiates a Rapid and Specific Transcriptional Response during Surface Attachment. *J. Bacteriol.* **204**, e00086-22 (2022).

109. Maeda, H. *et al.* Purification and characterization of a biodegradable plastic-degrading enzyme from Aspergillus oryzae. *Appl. Microbiol. Biotechnol.* **67**, 778–788 (2005).
